# One hundred most cited articles related to Endoscopic retrograde cholangiopancreatography: A bibliometric analysis

**DOI:** 10.3389/fsurg.2022.1005771

**Published:** 2022-11-09

**Authors:** Xuan Xu, Lulu Guan, Yao Wu, Huajing Ke, Yuanbin Zhao, Pi Liu

**Affiliations:** ^1^ Department of Gastroenterology, The First Affiliated Hospital of Nanchang University, Nanchang, China; ^2^ First Clinical Medical College, Nanchang University, Nanchang, China; ^3^ Second Clinical Medical College, Nanchang University, Nanchang, China; ^4^ Department of Gastroenterology, The People’s Hospital of Longhua, Shenzhen, China

**Keywords:** ERCP, bibliometric analysis, visualization, post-ERCP pancreatitis, top-cited

## Abstract

**Background:**

Endoscopic retrograde cholangiopancreatography (ERCP) has developed over the past few decades into a reliable technology for diagnostic and therapeutic purposes. Through a bibliometric analysis, this research attempted to evaluate the characteristics of the top 100 articles on ERCP that had the most citations.

**Methods:**

We extracted pertinent publications from the Web of Science Core Collection (WoSCC) on July 9, 2022. The top 100 ERCP articles with the most citations were identified and analyzed. The following data were extracted: publication year, country/region, organization, total citation times, annual citation times, research type and research field, etc. To implement the network’s visual analysis, a bibliographic coupling network based on keywords was built using the VOSviewer 1.6.17 program.

**Results:**

The journal with the most publications were *GASTROINTESTINAL ENDOSCOPY*, with 45 articles. Most of the top 100 articles came from the United States (*n* = 47) and Italy (*n* = 14). Indiana University and the University of Amsterdam were among the most important institutions in ERCP research. ML Freeman of the University of Minnesota contributed the highest number (*n* = 9) and the most highly cited paper. The age of the paper and article type is closely related to citation frequency. Of the 100 most-cited articles, clinical application in the field of ERCP has focused on three aspects: diagnosis, treatment, and complications. Clinical use of ERCP has shifted from diagnosis to treatment. Post-ERCP pancreatitis is the focus of attention, and the clinical application of technically complex therapeutic ERCP is the future development trend.

**Conclusion:**

This study lists the most influential articles in ERCP by exposing the current state of the field, and showing the evolution of research trends to provide perspective for the future development of ERCP.

## Introduction

Endoscopic retrograde cholangiopancreatography (ERCP), developed in the late 1960s and first described by American researcher McCune et al. in 1968, is a noninvasive or minimally invasive technique for the diagnosis and treatment of hepatobiliary and pancreatic illnesses (1, 2). They performed the world’s first intubation of the duodenal papilla using a side-view fiberoptic duodenoscope. Although the intubation success rate was only 25% at the time, it opened a new field of diagnosing and treating biliary and pancreatic diseases (1). ERCP was initially developed as a diagnostic aid, with the operator injecting a contrast agent to understand lesions in the biliopancreatic duct. In the past half-century, with the continuous improvement of operation technology, endoscopy, and its accessory instruments have developed rapidly. ERCP has gradually become an essential interventional therapy for biliary and pancreatic diseases (3, 4). ERCP-related technologies include endoscopic sphincterotomy (EST), endoscopic naso-biliary drainage (ENBD), endoscopic retrograde biliary drainage (ERBD), endoscopic papillary balloon dilation (EPBD), endoscopic naso-pancreatic drainage (ENPD), endoscopic retrograde pancreatic drainage (ERPD), transoral choledochoscopy and treatment, transoral pancreatoscopy, integrable duct ultrasound (IDUS), Spyglass, etc. The development of these techniques has been widely used to treat pancreatic disorders, bile duct strictures, and stones in the bile duct. Even though ERCP has grown to be an effective clinical treatment, complications and adverse events that follow ERCP still exist and may significantly affect patients’ morbidity and, very rarely, fatality (5). Pancreatitis, bleeding, cholecystitis, infection, and intestinal perforation are typical post-ERCP complications. The most frequent complication following ERCP is post-ERCP pancreatitis (PEP), whose incidence varies from 3.5% to 9.7%, as reported in meta-analyses and approaches 15% in high-risk patients (6, 7).

Bibliometric analysis is a popular statistical method used to evaluate the characteristics of publications in a specific field. We can quickly obtain information in this field through quantitative and qualitative analysis, evaluate research hotspots, and explore research trends. Since the first bibliometric analysis was published in 1987, many bibliometric analyses have been published recently in various medical fields, such as Endoscopic ultrasound (EUS), liver cancer, and pancreatic neuroendocrine tumors (8–11). Despite increasing research in the ERCP field, nothing is known about the generation of scientific knowledge in this area, and a bibliometric analysis has not yet been published. Therefore, we selected the top 100 most-cited (T100) articles from the Web of Science (WOS) database to provide a bibliometric perspective for the study of ERCP and to reveal the development trend of the discipline.

## Materials and methods

The Science Citation Index (SCI-Expanded) of the Web of Science Core Collection (WoS-CC) database (Clarivate Analytics, United States) is thought to be the most suitable database for bibliometric analysis and was used to conduct a thorough literature search. Ethics Committee approval was not required for this study as it did not involve intervention or data collection in animal or clinical trials. In the SCI-Expanded of WoS-CC, we created search terms based on MESH subject terms and synonyms, as follows with no language, publication type or publication time limit: TS = (“Cholangiopancreatography, Endoscopic Retrograde” OR “Retrograde Cholangiopancreatography, Endoscopic” OR “Cholangiopancreatographies, Endoscopic Retrograde” OR “Endoscopic Retrograde Cholangiopancreatographies” OR “Retrograde Cholangiopancreatographies, Endoscopic” OR “Endoscopic Retrograde Cholangiopancreatography” OR “ERCP”). To prevent changes in the online activity of articles, all data were collected on July 9, 2022.

The retrieved literature is sorted in descending order in the database according to the number of citations. Two researchers (XX and GLL) independently reviewed the abstract or full text to ensure that only studies focusing on ERCP were included in the subsequent analysis. Those that mentioned ERCP only in passing were excluded until the T100 articles were identified. The third researcher (LP) shall settle the differences between the two researchers through negotiation. Relevant information about the T100 articles, including publication date, citation counts, annual citations (total citations/the number of years since publication), author, journal of publication, country of origin, institution, study type, and research field, were extracted to Microsoft Excel 2019. The journal impacts factor 2021 (IF 2021) and quartile were from 2021 Journal Citation Reports (12). In addition, we also downloaded “Full record and cited references” in plain text format and used them in the analysis of bibliometric analysis tools.

VOSviewer 1.6.17 software was used to establish the bibliometric network’s author and keyword co-occurrence map. We also detected keyword hotspots and trends by time of appearance. In the network co-occurrence graph, nodes represent elements such as authors or keywords. The size of nodes represents the frequency of element occurrence, the line between nodes indicates the cooperative relationship, and the closer the distance between nodes indicates the closer relationship. Qualitative data were presented as the frequency in percentage. Quantitative data were presented as average or median (first quartile [Q1], third quartile [Q3]) after being tested for normality by the Shapiro-Wilk test. The Pearson and Spearman correlation was used to evaluate bivariate correlation, and *P* < 0.05 was considered statistically significant. All data were statistically analyzed using IBM SPSS version 26.0 software (IBM Corp., Armonk, NY, United States).

## Results

The main characteristics of the T100 articles are shown in Supplementary Table S1 (3, 5, 7, 9, 13–108). A total of 16,781 publications related to ERCP were initially retrieved, ranked in descending order of citation frequency. After screening, we identified the T100 articles. All 100 articles were published in English. There were 28,129 citations for T100 articles, with a median citation count of 218 (range 159–1,925). Among the T100 articles, Surprisingly, the most cited article was 1,925 times, well ahead of the next most cited paper, 898 times (28, 38). The annual citations of T100 articles varied from 5.82 to 71.3 times, with a median citation count of 11.92.

### Distribution of articles by years of publication

**Figure 1:**
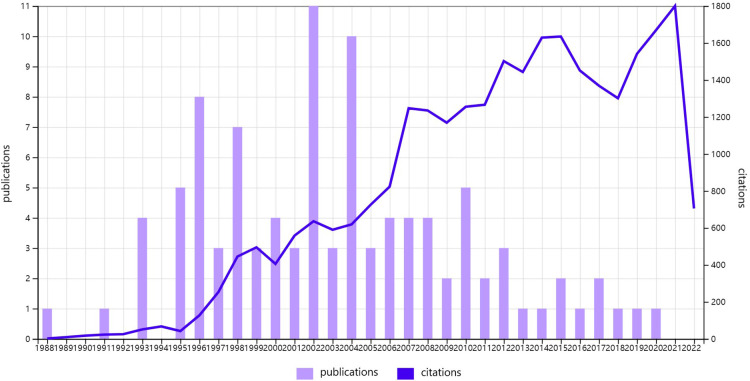
The publication time and citation distribution of the T100 articles in ERCP.

**Figure 2:**
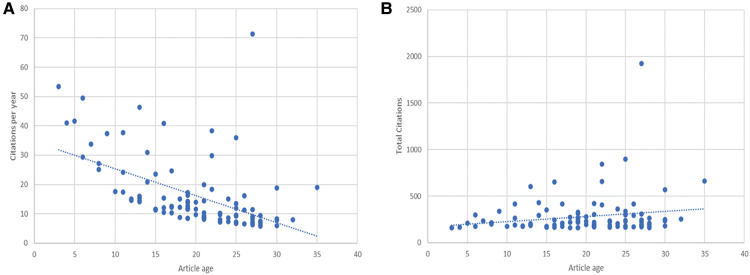
Citations analysis. (**A**) Correlation between article age and average annual citations since publication. (**B**) Correlation between article age and total citations since publication.

The T100 articles in this field were published in the 32 years from 1988 to 2020. In chronological order, we noticed that 68% of the papers were published after 2000, and 48% were published between 2000 and 2010 (Figure 1). 2002 had the highest number of publications (*n* = 11), followed by 2004 (*n* = 10). The oldest article was by Neoptolemos JP et al., published in 1988 (13). The most recent article was published in 2020 by Dumonceau et al. (5). Moreover, there was an inverse correlation between annual citations since publication and article age (*ρ* = −0.638; *P* < 0.001) (Figure 2A). However, there was no correlation between the age of the paper and total citations (TC) (*P* = 0.174) (Figure 2B).

### Distribution of the institution and country

**Figure 3:**
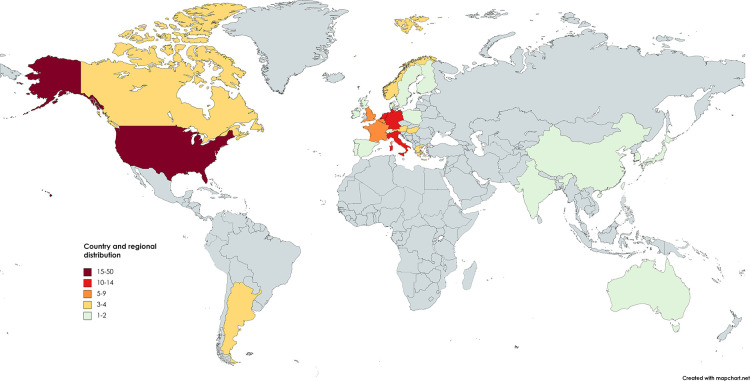
World map for the geographical distribution of the T100 articles.

Twenty-nine countries or regions contributed to The T100 articles (Figure 3). The USA contributed the most publications (47 papers) and the highest total citations (TC), followed by Italy (14 documents) and Germany (12 documents). Thirteen countries contributed three or more articles (Table 1). In terms of research institutions, Indiana University and the University of Amsterdam contributed the most papers (both eight papers) (Table 2). Moreover, Indiana University leads in TC (*n* = 3870) and mean citations per article (*n* = 483.8). The University of Minnesota in the United States and the Università Vita-Salute San Raffaele in Italy tied for third place with six articles each.

**Table 1:** countries or regions published at least three articles**.** TC = Total citations.

Country	Publications	TC	Mean citations per article
USA	47	13,400	285.11
ITALY	14	4681	334.36
GERMANY	12	2665	222.08
NETHERLANDS	10	2422	242.2
ENGLAND	9	2519	279.89
FRANCE	9	1980	220
BELGIUM	8	1686	210.75
CANADA	4	2525	631.25
ARGENTINA	4	945	236.25
GREECE	4	768	192
HUNGARY	4	768	192
AUSTRIA	3	733	244.33
NORWAY	3	560	186.67

**Table 2:** Institution published at least four articles. TC = Total citations.

Institution	documents	TC	Mean citations per article	Country
Indiana University	8	3870	483.75	USA
University of Amsterdam	8	1969	246.13	Netherlands
University of Minnesota	6	1995	332.5	USA
Università Vita-Salute San Raffaele	6	1286	214.33	Italy
Medical University of South Carolina	5	1718	343.6	USA
University of Michigan	5	1377	275.4	USA
Gedyt Endoscopy Center	4	945	236.25	Argentina
Universite libre de Bruxelles	4	945	236.25	Netherlands
Università Cattolica del Sacro Cuore	4	923	230.75	Italy
Hop edouard herriot	4	801	200.25	France

### Analysis of authors

**Figure 4:**
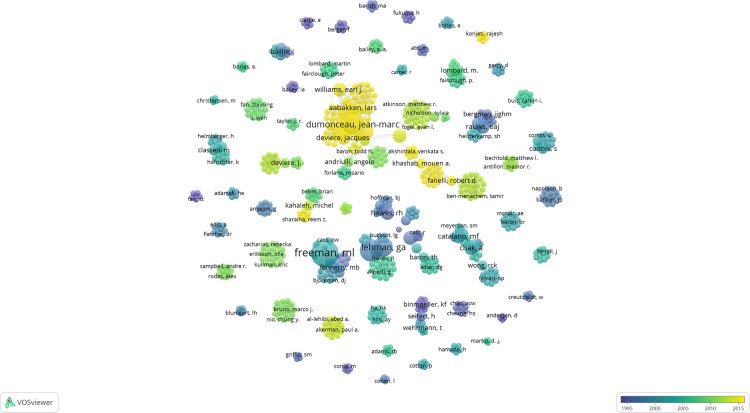
VOSviewer co-authorship map illustrating author density and the existence of clusters among all authors of the 100 most-cited articles. Each node represents a different author, and the node size is proportional to the number of publications. The color represents the average year the author published.

A total of 733 authors contributed to the T100 articles, of which nine published at least five (Table 3). The list was led by Freeman ML and Mariani A, who wrote 9 of the T100 articles each. The nine papers by Freeman ML et al. from the University of Minnesota were cited 4,535 times in the T100 articles. At the same time, he has seven articles as a corresponding author, which is the most. A network was constructed of the co-authors of the T100 articles (Figure 4). As can be seen from this figure, many groups have formed among the authors, and there seems to be a lack of collaboration between groups. The figure shows that Freeman ML seems to be the most prominent author, but the team with Dumonceau JM as the core has been outstanding in recent years.

**Table 3:** First, senior, or corresponding authors who have published at least five T100 articles. TC = Total citations.

				Author position				
Author	Number of papers	TC	Mean citations per article	First-author	Correspondent author	others	Affiliation	country
Freeman ML	9	4535	503.9	0	7	2	Univ Minnesota	USA
Mariani A	9	2533	281.4	0	0	9	Univ Vita Salute San Raffaele	Italy
Sherman S	7	3701	528.7	0	3	4	Indiana Univ	USA
Dumonceau JM	7	1504	214.9	0	5	2	Hop Civil Marie Curie	Belgium
Testoni PA	6	1900	316.7	0	2	4	Univ Vita Salute San Raffaele	Italy
Lehman GA	6	1776	296	0	0	6	Indiana Univ	USA
Deviere J	6	1361	226.8	0	0	6	Univ Libre Bruxelles	Belgium
Williams EJ	5	1359	271.8	3	0	2	Royal Bournemouth Hosp	England
Tringali A	5	960	192	0	0	5	Catholic Univ	Italy

### Analysis of journals

A total of 15 journals with an IF between 3.243 and 202.731 published the T100 influential articles (Table 4). The *Lancet* had the highest IF (IF = 202.731). *Gastrointestinal Endoscopy* not only has the most significant number of T100 articles published (*n* = 45) but also leads the field with 11,305 citations. The *New England Journal of Medicine* had the highest average number of citations per article (TC/publications 705.8), followed by the *Lancet* (TC/publications 364.0) and the *American Journal of Gastroenterology* (TC/publications 360.6). Most of these journals were in the first quartile (Q1) of their corresponding disciplines, except for one journal that was in the second (Q2) and two journals that were in the three (Q3). In the T100 influential articles, there is a positive correlation between the TC/publications and corresponding journal IF (*ρ* = 0.745; *P* = 0.001). However, the corresponding journal IF is unrelated to TC or the number of T100 articles published in each journal (*P* > 0.05).

**Table 4:** Journals of top-cited articles.TC = total citations, IF = impact factor. *In 2013 ARCHIVES OF SURGERY changed to JAMA Surgery.

Journals	Publications	TC	TC/publications	IF2021	Quartile (2021)
GASTROINTESTINAL ENDOSCOPY	45	11,305	251.2	10.396	Q1
ENDOSCOPY	15	3418	227.9	9.776	Q1
AMERICAN JOURNAL OF GASTROENTEROLOGY	8	2885	360.6	12.045	Q1
NEW ENGLAND JOURNAL OF MEDICINE	6	4235	705.8	176.079	Q1
GUT	5	1073	214.6	31.793	Q1
LANCET	4	1456	364.0	202.731	Q1
RADIOLOGY	4	842	210.5	29.146	Q1
ANNALS OF SURGERY	3	774	258.0	13.787	Q1
GASTROENTEROLOGY	3	734	244.7	33.883	Q1
*ARCHIVES OF SURGERY	2	365	182.5	16.689	Q1
ANNALS OF INTERNAL MEDICINE	1	238	238	51.598	Q1
COCHRANE DATABASE OF SYSTEMATIC REVIEWS	1	175	175	12.008	Q1
DIGESTIVE DISEASES AND SCIENCES	1	163	163	3.487	Q3
LIVER TRANSPLANTATION	1	212	212	6.112	Q2
PANCREAS	1	254	254	3.243	Q3

### Distribution of study types and topics

The most common type of study was prospective study (*n* = 30), followed by retrospective study (*n* = 24), randomized controlled trials (RCT) (*n* = 18), guideline (*n* = 11), systematic reviews (*n* = 10), review (*n* = 4), Case report (*n* = 2) and Conference papers (*n* = 1). Treatment was the most studied subtopic (*n* = 36), followed by Complications (*n* = 32), diagnosis (*n* = 20), EUS-guided interventional therapy when ERCP fails (*n* = 6). Among the 20 articles of diagnostic type, 12 were comparative studies of ERCP and EUS, CT, MRI/MRCP. Therefore, the comparison of ERCP and its competing technologies in diagnosis and treatment is also the focus of researchers. According to the number of citations of various articles, prospective studies have the highest average number of citations per paper. However, the annual citations for guidelines are much higher than those of other publication types, but because this category of articles has mostly been published in recent years, there has been insufficient time to accumulate citations (Table 5).

**Table 5:** Types of documents. * Expressed in the median (first quartile [Q1], third quartile [Q3]).

Rank	Study type	Publications	TC	Mean citations per article	annual citations *
1	*P*rospective study	30	10,166	338.9	9.65 (8.22;15.58)
2	Retrospective study	24	5112	213.0	8.88 (7.58;12.15)
3	Randomized controlled Trials	18	5821	323.4	12.64 (10.83;18.90)
4	Guideline	11	2693	244.8	33.71 (17.45;41.60)
5	Systematic reviews	10	2512	251.2	14.29 (11.60;27.68)
6	Review	4	984	246.0	10.42 (8.12;16.03)
7	Case report	2	668	334.0	13.84, 18.41
8	Conference papers	1	173	173.0	8.24

**Figure 5:**
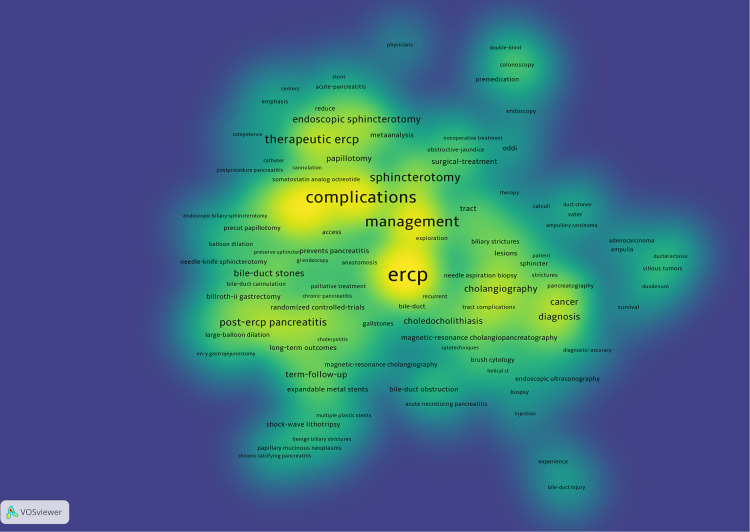
Heat map of the 100 most-cited articles in keywords. The darker the color, the more times the keyword appears.

**Figure 6:**
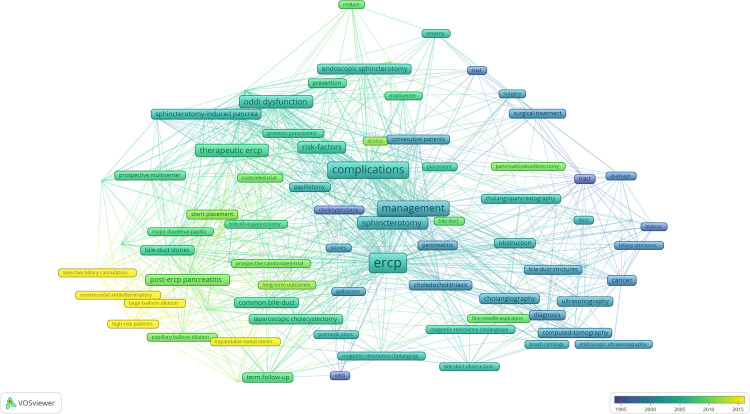
Overlay visualization map showing trends of keyword frequency over time. Colors were assigned according to the average year in which keywords appeared in articles.

In VosViewer, we merged synonyms and different variants of the same keyword (Supplementary document) and extracted 301 keywords. VOSViewer heat map detailed the keywords relationships from the T100 articles (Figure 5). Keywords that appear more frequently include “ERCP” 43 times, “complications” 30 times, “management” 25 times, “oddi dysfunction” 17 times, “sphincterotomy” 16 times, “therapeutic ERCP” 16 times, “biliary sphincterotomy” 13 times,” risk-factors” 13 times,” post-ERCP pancreatitis” 11 times and “endoscopic sphincterotomy” 10 times. From the heat map, we could find that the current attention of ERCP mainly focuses on: “diagnosis,” “treatment,” and “complications.” Moreover, 81 keywords with a minimum number of occurrences of three were analyzed; an overlay visualization map shows how the trends of keywords change over years (Figure 6). For example, “diagnosis,” “computed tomography,” and “cholecystectomy” appeared before 2000 and then” complications”, “therapeutic ERCP”,” prospective multicenter “, “controlled trial” and “long-term outcome” began to appear. The top keywords in recent years were “high-risk patients”, “expandable metal stents”, “selective biliary cannulation”, “large-balloon dilation”, and “nonsteroidal antiinflammatory drugs”.

## Discussion

A large number of studies have promoted the field of ERCP. Advances in the Internet have made it easy for researchers to obtain the latest research results, but this has posed a challenge for researchers to get valuable and high-quality research from many resources. Citation analysis can qualitatively and quantitatively analyze the research status and development history of a specific field, which helps identify classical research and high-impact journals (109). Therefore, this study identified highly cited literature related to ERCP through bibliometrics and evaluated their characteristics to have a deeper understanding of this field.

The number of citations is an important index to evaluate the influence of a paper. In the current research, all top 100 papers have been cited at least 100 times, and the citation frequency of the documents is between 159 and 1925. Although the inclusion criteria were not identical, it was found that the number of citations of ERCP was higher than that of other endoscopes, such as bronchoscopy (*n* = 196–731) and ankle arthroscopy (*n* = 56–225), indicating that ERCP is a hot topic (110, 111). The top-ranked publication, “Complications of endoscopic biliary sphincterotomy,” had a total of 1925 citations; it was published in the *NEW ENGLAND JOURNAL OF MEDICINE* in 1996 by Freeman et al. (28). Freeman et al. conducted a prospective cohort study of sphincterotomy at 16 institutions in the United States and 1 in Canada. In their paper, 2,347 patients undergoing biliary sphincterotomy were analyzed, and the risk factors of ERCP complications were summarized, providing a reference for future studies (28).

The papers’ geographic distribution was also made clear. Most T100 ERCP research came from nations and organizations in Western Europe and North America. Among the T100 articles, four of the top ten publishing institutions are from the United States. Indiana University from the USA ranked first in the number of articles and TC. This indicates that the USA is in a leading position in ERCP research. As a country with the highest GDP in the world, the United States has top medical research institutions and researchers. According to statistics, the USA led the world in scientific research spending from 1981 to 2020, which might explain why the USA also leads in several other areas of medicine (112–116). In addition, we found that every continent except Africa participated in the T100 articles, indicating that ERCP is widely used and researched. The most significant scholar on ERCP, Freeman ML, authored 9 of the top 100 articles with the most 4,535 TC. Freeman ML’s studies focused on post-ERCP complications and were published between 1996 and 2006 (28, 49, 55, 62, 69–71, 77, 79). Complications of ERCP are hot topics for endoscopists and are often cited as such. Our analysis revealed that Freeman’s article entitled “Complications of endoscopic biliary sphincterotomy” was the most cited paper (*n* = 1,925) (28). We found that Dumonceau JM is one of the most prolific authors to emerge in recent years, publishing between 2010 and 2020. He has co-authored seven European Society of Gastrointestinal Endoscopy (ESGE) Guidelines, five of which are the corresponding author (3, 5, 92, 100, 103, 105, 108).

We found no significant correlation between IF and the number of T100 articles in the corresponding journals. This could be explained by the diversity of publications’ fields of expertise or subject matter. We note that 60% of the papers were published on *GASTROINTESTINAL ENDOSCOPY* (*n* = 45) and *ENDOSCOPY* (*n* = 15) with IF 10.396 and 9.776, respectively. This indicates that ERCP-related studies are more likely to be published in professional endoscopy journals. Generally, the well-recognized: ESGE consensus guidelines for ERCP are published by *ENDOSCOPY*. In addition to the specific journals on digestive endoscopy, some of the top journals in the medical specialty, such as The *New England Journal of Medicine* and *LANCET*, also played an important role in this analysis. Ten papers were published in prestigious academic journals with high impact factors in these two medical fields. Although their T100 articles are small in number, they lead in citations per article. The IFs of the 15 journals that published the T100 articles were all >3.0, demonstrating the vitality and significance of ERCP research. The most cited ERCP papers were almost published in US and UK journals, most of them with high IF. These esteemed journals have strong reputations and widespread influence, which draw readers and citations. So high-performing researchers may be more willing to submit high-quality papers to these journals, thus maintaining a high IF (115).

Notably, two case reports received 668 citations (50, 71). They described EUS-guided biliary puncture and drainage (EUS-BD) as an important remedial treatment for the failure of ERCP in benign and malignant biliary ducts. Patients with unresectable malignant distal biliary obstruction were randomized to EUS-BD or ERCP in an RCT. The results showed no statistically significant difference in clinical and technical success between EUS-BD and ERCP. EUS-BD was associated with fewer postoperative complications and higher quality of life (116). However, more research still needs to research whether EUS-BD can replace ERCP as a therapeutic strategy for newly diagnosed patients. In addition, prospective studies and RCTs received the highest average citations per paper (338.9 and 323.4, respectively). Considering the influence of publication time on the number of citations, we calculate the average annual citations. The results showed that guidelines and systematic reviews had the highest average annual citations. Therefore, this embodies the idea of evidence-based medicine, where studies with higher levels of evidence are more likely to be cited.

In terms of research hotspots, besides “ERCP”, “complications” were the most frequent keywords (*n* = 30). The three most cited articles were all prospective studies of ERCP complications (28, 38, 49). The most recent of the T100 articles is the guidelines on complications of ERCP published by ESGE in 2020 (5). Therefore, complications are undoubtedly the most concern for ERCP, with PEP being the most concerned. Given the high incidence of PEP and the health care costs associated with it, many studies have been conducted to prevent PEP. Prophylactic rectal indomethacin dramatically decreased the incidence and severity of PEP in patients at high risk for this complication, according to one RCT trial cited 414 times (101). A recent meta-analysis confirmed these results, but further research is needed on additional prevention methods, including pancreatic stents and intravenous fluids (117).

Regarding research trends, we can clearly see the time evolution of ERCP from diagnosis to treatment (Figure 6). Our overlay visualization map clearly illustrates the rise of “therapeutic ERCP” in the 2000s (Figure 6). ERCP was initially developed in 1968 as a diagnostic tool. In 1974, Drs Meinhard Classen in Germany and Keiichi Kawai in Japan simultaneously undertook the first biliary sphincterotomy. Since then, ERCP has evolved from a diagnostic to a therapeutic tool (118). With the development of imaging, including the widespread use of noninvasive tests such as computed tomography, magnetic resonance cholangiopancreatography (MRCP), and EUS, diagnostic ERCP has been partially replaced, making its use less and less (119, 120). Although MRCP can identify a range of pancreatic and biliary diseases, including biliary obstruction (121), evaluation of biliary pathologies (122), and choledocholithiasis (123), some studies have pointed out that MRCP cannot completely replace ERCP in the diagnosis of pancreatic and biliary diseases, especially mild bile duct lesions (124, 125). Because of the invasive nature and risk of ERCP, purely diagnostic ERCP has been gradually reduced and is currently mainly used in patients whose etiology cannot be determined by noninvasive tests. Combined with recent keywords, we found that the use of a nonsteroidal anti-inflammatory drug to prevent PEP in high-risk patients, endoscopic large balloon dilation of papillary sphincter for common bile duct calculi, and self-expandable metal stents for benign and malignant biliary obstruction and selective biliary cannulation are recent research hotspots. Thus, the prospect of ERCP lies in the development of new treatment techniques and how to prevent the complications of the procedure. Admittedly, the development of some competing technologies has challenged the clinical application of ERCP, but therapeutic ERCP with increased technical complexity is the way forward in the future.

Although our results provide valuable information, like other bibliometric research, our study has several limitations. First, evaluating the literature only by the number of citations is not comprehensive. For example, the latest important literature does not have enough time to accumulate citations. Second, although we did our best to conduct a comprehensive search, ERCP-related literature may still be missing. Another limitation of this study is that open-access vs. subscription-based journals were not filtered separately during the WOS search. This would seemingly be an impactful factor in how often these articles are being cited. Finally, we only used the WOS database to search literature, and literature from other databases such as Scopus and Google Scholar may be omitted. However, WOS is the most suitable database for bibliometric analysis, which contains the most comprehensive citation information.

## Conclusions

To the best of our knowledge, this is the first bibliometric study of the T100 articles on ERCP. The T100 articles ranged from 159 to 1,925 citations, with publication years ranging from 1988 to 2020. The number of articles published in 2002 was the highest. The USA was the most significant contributor, followed by Italy and Germany. Indiana University was the center of ERCP research. The paper’s age and article type are closely related to citation frequency. Among the T100 articles, clinical application in ERCP has focused on three aspects: diagnosis, treatment, and complications. PEP is the focus of attention, and more technically complex therapeutic ERCP will be further developed. In summary, this study identified the T100 ERCP articles with the highest citation frequency and analyzed their bibliometric characteristics, laying a foundation for further research.

## Data Availability

The original contributions presented in the study are included in the article/**Supplementary Material**, further inquiries can be directed to the corresponding author/s.

## References

[1] McCune WS Shorb PE Moscovitz H. Endoscopic cannulation of the ampulla of vater: a preliminary report. Ann Surg. (1968) 167(5):752–6. 10.1097/00000658-196805000-00013 5646296 PMC1387128

[2] Sanders DJ Bomman S Krishnamoorthi R Kozarek RA. Endoscopic retrograde cholangiopancreatography: current practice and future research. World J Gastrointest Endosc. (2021) 13(8):260–74. 10.4253/wjge.v13.i8.260 34512875 PMC8394185

[3] Manes G Paspatis G Aabakken L Anderloni A Arvanitakis M Ah-Soune P Endoscopic management of common bile duct stones: European society of gastrointestinal endoscopy (ESGE) guideline. Endoscopy. (2019) 51(5):472–91. 10.1055/a-0862-0346 30943551

[4] Vozzo CF Sanaka MR. Endoscopic management of pancreaticobiliary disease. Surg Clin North Am. (2020) 100(6):1151–68. 10.1016/j.suc.2020.08.006 33128885

[5] Dumonceau JM Kapral C Aabakken L Papanikolaou IS Tringali A Vanbiervliet G ERCP-related adverse events: European society of gastrointestinal endoscopy (ESGE) guideline. Endoscopy. (2020) 52(2):127–49. 10.1055/a-1075-4080 31863440

[6] Kochar B Akshintala VS Afghani E Elmunzer BJ Kim KJ Lennon AM Incidence, severity, and mortality of post-ERCP pancreatitis: a systematic review by using randomized, controlled trials. Gastrointest Endosc. (2015) 81(1):143–9.e9. 10.1016/j.gie.2014.06.045 25088919

[7] Andriulli A Loperfido S Napolitano G Niro G Valvano MR Spirito F Incidence rates of post-ERCP complications: a systematic survey of prospective studies. Am J Gastroenterol. (2007) 102(8):1781–8. 10.1111/j.1572-0241.2007.01279.x 17509029

[8] Xu G Jin B Xian X Yang H Zhao H Du S Evolutions in the management of hepatocellular carcinoma over last 4 decades: an analysis from the 100 most influential articles in the field. Liver Cancer. (2021) 10(2):137–50. 10.1159/000513412 33977090 PMC8077437

[9] Shi H Chen H Qian B Huang Z Tan P Peng Y The 100 most cited articles on pancreatic neuroendocrine tumors from 2000 to 2020: a bibliometric analysis. Jpn J Clin Oncol. (2021) 52(3):251–9. 10.1093/jjco/hyab205 34954797

[10] Chen X He H Chen X Chen X Wen Z Xu M A bibliometric analysis of publications on endoscopic ultrasound. Front Med (Lausanne). (2022) 9:869004. 10.3389/fmed.2022.869004 35425770 PMC9002052

[11] Garfield E. 100 citation-classics from the journal-of-the-American-medical-association. JAMA. (1987) 257(1):52–9. 10.1001/jama.257.1.52 3537352

[12] https://clarivate.com/webofsciencegroup/solutions/journal-citation-reports/.

[13] Neoptolemos JP London NJ James D Carrlocke DL Bailey IA Fossard DP. Controlled trial of urgent endoscopic retrograde cholangiopancreatography and endoscopic sphincterotomy versus conservative treatment for acute-pancreatitis due to gallstones. Lancet. (1988) 2(8618):979–83. 10.1016/s0140-6736(88)90740-4 2902491

[14] Sherman S Lehman GA. ERCP-scopic and endoscopic sphincterotomy-induced pancreatitis. Pancreas. (1991) 6(3):350–67. 10.1097/00006676-199105000-00013 1713676

[15] Binmoeller KF Boaventura S Ramsperger K Soehendra N. Endoscopic snare excision of benign adenomas of the papilla of vater. Gastrointest Endosc. (1993) 39(2):127–31. 10.1016/s0016-5107(93)70051-6 8495831

[16] Fan ST Lai ECS Mok FPT Lo CM Zheng SS Wong J. Early treatment of acute biliary pancreatitis by endoscopic papillotomy. N Engl J Med. (1993) 328(4):228–32. 10.1056/nejm199301283280402 8418402

[17] Nealon WH Thompson JC Fischer JE Andersen D. Progressive loss of pancreatic function in chronic-pancreatitis is delayed by main pancreatic duct decompression—a longitudinal prospective analysis of the modified puestow procedure. Ann Surg. (1993) 217(5):458–68. 10.1097/00000658-199305010-00005 8489308 PMC1242821

[18] Wiersema MJ Hawes RH Lehman GA Kochman ML Sherman S Kopecky KK. Prospective evaluation of endoscopic ultrasonography and endoscopic retrograde cholangiopancreatography in patients with chronic abdominal-pain of suspected pancreatic origin. Endoscopy. (1993) 25(9):555–64. 10.1055/s-2007-1010405 8119204

[19] Binmoeller KF Jue P Seifert H Nam WC Izbicki J Soehendra N. Endoscopic pancreatic stent drainage in chronic pancreatitis and a dominant stricture: long-term results. Endoscopy. (1995) 27(9):638–44. 10.1055/s-2007-1005780 8903975

[20] Macmathuna P White P Clarke E Merriman R Lennon JR Crowe J. Endoscopic balloon sphincteroplasty (papillary dilation) for bile-duct stones—efficacy, safety, and follow-up in 100 patients. Gastrointest Endosc. (1995) 42(5):468–74. 10.1016/s0016-5107(95)70052-8 8566640

[21] Minami A Nakatsu T Uchida N Hirabayashi S Fukuma H Morshed SA Papillary dilation vs sphincterotomy in endoscopic removal of bile duct stones—a randomized trial with manometric function. Dig Dis Sci. (1995) 40(12):2550–4. 10.1007/bf02220440 8536511

[22] Ponchon T Gagnon P Berger F Labadie M Liaras A Chavaillon A Value of endobiliary brush cytology and biopsies for the diagnosis of malignant bile-duct stenosis—results of a prospective-study. Gastrointest Endosc. (1995) 42(6):565–72. 10.1016/s0016-5107(95)70012-9 8674929

[23] Pugliese V Conio M Nicolo G Saccomanno S Gatteschi B. Endoscopic retrograde forceps biopsy and brush cytology of biliary strictures—a prospective-study. Gastrointest Endosc. (1995) 42(6):520–6. 10.1016/s0016-5107(95)70004-8 8674921

[24] Bergman J vander Mey S Rauws EAJ Tijssen JGP Gouma DJ Tytgat GNJ Long-term follow-up after endoscopic sphincterotomy for bile duct stones in patients younger than 60 years of age. Gastrointest Endosc. (1996) 44(6):643–9. 10.1016/s0016-5107(96)70045-7 8979051

[25] Bret PM Reinhold C Taourel P Guibaud L Atri M Barkun AN. Pancreas divisum: evaluation with MR cholangiopancreatography. Radiology. (1996) 199(1):99–103. 10.1148/radiology.199.1.8633179 8633179

[26] Cavallini G Tittobello A Frulloni L Masci E Mariani A DiFrancesco V Gabexate for the prevention of pancreatic damage related to endoscopic retrograde cholangiopancreatography. N Engl J Med. (1996) 335(13):919–23. 10.1056/nejm199609263351302 8786777

[27] Chan YL Chan ACW Lam WWM Lee DWH Chung SSC Sung JJY Choledocholithiasis: comparison of mr cholangiography and endoscopic retrograde cholangiography. Radiology. (1996) 200(1):85–9. 10.1148/radiology.200.1.8657949 8657949

[28] Freeman ML Nelson DB Sherman S Haber GB Herman ME Dorsher PJ Complications of endoscopic biliary sphincterotomy. N Engl J Med. (1996) 335(13):909–18. 10.1056/nejm199609263351301 8782497

[29] Jowell PS Baillie J Branch MS Affronti J Browning CL Bute BP. Quantitative assessment of procedural competence—a prospective study of training in endoscopic retrograde cholangiopancreatography. Ann Intern Med. (1996) 125(12):983–9. 10.7326/0003-4819-125-12-199612150-00009 8967710

[30] Soto JA Barish MA Yucel EK Siegenberg D Ferrucci JT Chuttani R. Magnetic resonance cholangiography: comparison with endoscopic retrograde cholangiopancreatography. Gastroenterology. (1996) 110(2):589–97. 10.1053/gast.1996.v110.pm8566608 8566608

[31] Wiersema MJ Sandusky D Carr R Wiersema LM Erdel WC Frederick PK. Endosonography-guided cholangiopancreatography. Gastrointest Endosc. (1996) 43(2):102–6. 10.1016/s0016-5107(06)80108-2 8635700

[32] Bergman J Rauws EAJ Fockens P vanBerkel AM Bossuyt PMM Tijssen JGP Randomised trial of endoscopic balloon dilation versus endoscopic sphincterotomy for removal of bileduct stones. Lancet. (1997) 349(9059):1124–9. 10.1016/s0140-6736(96)11026-6 9113010

[33] Folsch UR Nitsche R Ludtke R Hilgers RA Creutzfeldt W. Early ERCP and papillotomy compared with conservative treatment for acute biliary pancreatitis. N Engl J Med. (1997) 336(4):237–42. 10.1056/nejm199701233360401 8995085

[34] Mansfield JC Griffin SM Wadehra V Matthewson K. A prospective evaluation of cytology from biliary strictures. Gut. (1997) 40(5):671–7. 10.1136/gut.40.5.671 9203949 PMC1027173

[35] Canto MIF Chak A Stellato T Sivak MV. Endoscopic ultrasonography versus cholangiography for the diagnosis of choledocholithiasis. Gastrointest Endosc. (1998) 47(6):439–48. 10.1016/s0016-5107(98)70242-1 9647366

[36] Catalano MF Lahoti S Geenen JE Hogan WJ. Prospective evaluation of endoscopic ultrasonography, endoscopic retrograde pancreatography, and secretin test in the diagnosis of chronic pancreatitis. Gastrointest Endosc. (1998) 48(1):11–7. 10.1016/s0016-5107(98)70122-1 9684658

[37] Cellier C Cuillerier E Palazzo L Rickaert F Flejou JF Napoleon B Intraductal papillary and mucinous tumors of the pancreas: accuracy of preoperative computed tomography, endoscopic retrograde pancreatography and endoscopic ultrasonography, and long-term outcome in a large surgical series. Gastrointest Endosc. (1998) 47(1):42–9. 10.1016/s0016-5107(98)70297-4 9468422

[38] Loperfido S Angelini G Benedetti G Chilovi F Costan F De Berardinis F Major early complications from diagnostic and therapeutic ERCP: a prospective multicenter study. Gastrointest Endosc. (1998) 48(1):1–10. 10.1016/s0016-5107(98)70121-x 9684657

[39] Rhodes M Sussman L Cohen L Lewis MP. Randomised trial of laparoscopic exploration of common bile duct versus postoperative endoscopic retrograde cholangiography for common bile duct stones. Lancet. (1998) 351(9097):159–61. 10.1016/s0140-6736(97)09175-7 9449869

[40] Sahai AV Zimmerman M Aabakken L Tarnasky PR Cunningham JT van Velse A Prospective assessment of the ability of endoscopic ultrasound to diagnose, exclude, or establish the severity of chronic pancreatitis found by endoscopic retrograde cholangiopancreatography. Gastrointest Endosc. (1998) 48(1):18–25. 10.1016/s0016-5107(98)70123-3 9684659

[41] Tarnasky PR Palesch YY Cunningham JT Mauldin PD Cotton PB Hawes RH. Pancreatic stenting prevents pancreatitis after biliary sphincterotomy in patients with sphincter of oddi dysfunction. Gastroenterology. (1998) 115(6):1518–24. 10.1016/s0016-5085(98)70031-9 9834280

[42] Fletcher DR Hobbs MST Tan P Valinsky LJ Hockey RL Pikora TJ Complications of cholecystectomy: risks of the laparoscopic approach and protective effects of operative cholangiography—a population-based study. Ann Surg. (1999) 229(4):449–57. 10.1097/00000658-199904000-00001 10203075 PMC1191728

[43] Hochwald SN Burke EC Jarnagin WR Fong YM Blumgart LH. Association of preoperative biliary stenting with increased postoperative infectious complications in proximal cholangiocarcinoma. Arch Surg. (1999) 134(3):261–6. 10.1001/archsurg.134.3.261 10088565

[44] Wehrmann T Kokabpick S Lembcke B Caspary WF Seifert H. Efficacy and safety of intravenous propofol sedation during routine ERCP: a prospective, controlled study. Gastrointest Endosc. (1999) 49(6):677–83. 10.1016/s0016-5107(99)70281-6 10343208

[45] Adamek HE Albert J Breer H Weitz M Schilling D Riemann JF. Pancreatic cancer detection with magnetic resonance cholangiopancreatography and endoscopic retrograde cholangiopancreatography: a prospective controlled study. Lancet. (2000) 356(9225):190–3. 10.1016/s0140-6736(00)02479-x 10963196

[46] Jailwala J Fogel EL Sherman S Gottlieb K Flueckiger J Bucksot LG Triple-tissue sampling at ERCP in malignant biliary obstruction. Gastrointest Endosc. (2000) 51(4):383–90. 10.1016/s0016-5107(00)70435-4 10744806

[47] Pfau PR Kochman ML Lewis JD Long WB Lucey MR Olthoff K Endoscopic management of postoperative biliary complications in orthotopic liver transplantation. Gastrointest Endosc. (2000) 52(1):55–63. 10.1067/mge.2000.106687 10882963

[48] Stapfer M Selby RR Stain SC Katkhouda N Parekh D Jabbour N Management of duodenal perforation after endoscopic retrograde cholangiopancreatography and sphincterotomy. Ann Surg. (2000) 232(2):191–8. 10.1097/00000658-200008000-00007 10903596 PMC1421129

[49] Freeman ML DiSario JA Nelson DB Fennerty MB Lee JG Bjorkman DJ Risk factors for post-ERCP pancreatitis: a prospective, multicenter study. Gastrointest Endosc. (2001) 54(4):425–34. 10.1067/mge.2001.117550 11577302

[50] Giovannini M Moutardier V Pesenti C Bories E Lelong B Delpero JR. Endoscopic ultrasound-guided bilioduodenal anastomosis: a new technique for biliary drainage. Endoscopy. (2001) 33(10):898–900. 10.1055/s-2001-17324 11571690

[51] Masci E Toti G Mariani A Curioni S Lomazzi A Dinelli M Complications of diagnostic and therapeutic ERCP: a prospective multicenter study. Am J Gastroenterol. (2001) 96(2):417–23. 10.1111/j.1572-0241.2001.03594.x 11232684

[52] Cohen S Bacon BR Berlin JA Fleischer D Hecht GA Loehrer PJ National institutes of health state-of-the-science conference statement: ERCP for diagnosis and therapy, January 14–16, 2002. Gastrointest Endosc. (2002) 56(6):803–9. 10.1067/mge.2002.129875 12447289

[53] Draganov P Hoffman B Marsh W Cotton P Cunningham J. Long-term outcome in patients with benign biliary strictures treated endoscopically with multiple stents. Gastrointest Endosc. (2002) 55(6):680–6. 10.1067/mge.2002.122955 11979250

[54] Enns R Eloubeidi MA Mergener K Jowell PS Branch MS Pappas TM ERCP-related perforations: risk factors and management. Endoscopy. (2002) 34(4):293–8. 10.1055/s-2002-23650 11932784

[55] Freeman ML. Adverse outcomes of ERCP. Gastrointest Endosc. (2002) 56(6):S273–82. 10.1067/mge.2002.129028 12447281

[56] Harewood GC Wiersema MJ. Endosonography-guided fine needle aspiration biopsy in the evaluation of pancreatic masses. Am J Gastroenterol. (2002) 97(6):1386–91. 10.1016/s0002-9270(02)04133-3 12094855

[57] Horiuchi A Kawa S Hamano H Hayama M Ota H Kiyosawa K. ERCP features in 27 patients with autoimmune pancreatitis. Gastrointest Endosc. (2002) 55(4):494–9. 10.1067/mge.2002.122653 11923760

[58] Ponsioen CY Vrouenraets SME Prawirodirdjo W Rajaram R Rauws EAJ Mulder CJJ Natural history of primary sclerosing cholangitis and prognostic value of cholangiography in a dutch population. Gut. (2002) 51(4):562–6. 10.1136/gut.51.4.562 12235081 PMC1773389

[59] Rerknimitr R Sherman S Fogel EL Kalayci C Lumeng L Chalasani N Biliary tract complications after orthotopic liver transplantation with choledochocholedochostomy anastomosis: endoscopic findings and results of therapy. Gastrointest Endosc. (2002) 55(2):224–31. 10.1067/mge.2002.120813 11818927

[60] Rosch T Meining A Fruhmorgen S Zillinger C Schusdziarra V Hellerhoff K A prospective comparison of the diagnostic accuracy of ERCP, MRCP, CT, and EUS in biliary strictures. Gastrointest Endosc. (2002) 55(7):870–6. 10.1067/mge.2002.124206 12024143

[61] Vandervoort J Soetikno RM Tham TCK Wong RCK Ferrari AP Montes H Risk factors for complications after performance of ERCP. Gastrointest Endosc. (2002) 56(5):652–6. 10.1067/mge.2002.129086 12397271

[62] Wright BE Cass OW Freeman ML. ERCP in patients with long-limb roux-en-Y gastrojejunostomy and intact Papilla. Gastrointest Endosc. (2002) 56(2):225–32. 10.1067/mge.2002.126136 12145601

[63] Fazel A Quadri A Catalano MF Meyerson SM Geenen JE. Does a pancreatic duct stent prevent post-ERCP pancreatitis? A prospective randomized study. Gastrointest Endosc. (2003) 57(3):291–4. 10.1067/mge.2003.124 12612504

[64] Masci E Mariani A Curioni S Testoni PA. Risk factors for pancreatitis following endoscopic retrograde cholangiopancreatography: a meta-analysis. Endoscopy. (2003) 35(10):830–4. 10.1055/s-2003-42614 14551860

[65] Murray B Carter R Imrie C Evans S O’Suilleabhain C. Diclofenac reduces the incidence of acute pancreatitis after endoscopic retrograde cholanglopancreatography. Gastroenterology. (2003) 124(7):1786–91. 10.1016/s0016-5085(03)00384-6 12806612

[66] Baron TH Harewood GC. Endoscopic balloon dilation of the biliary sphincter compared to endoscopic biliary sphincterotomy for removal of common bile duct stones during ERCP: a metaanalysis of randomized, controlled trials. Am J Gastroenterol. (2004) 99(8):1455–60. 10.1111/j.1572-0241.2004.30151.x 15307859

[67] Catalano MF Linder JD Chak A Sivak MV Raijman I Geenen JE Endoscopic management of adenoma of the major duodenal papilla. Gastrointest Endosc. (2004) 59(2):225–32. 10.1016/s0016-5107(03)02366-6 14745396

[68] Christensen M Matzen P Schulze S Rosenberg J. Complications of ERCP: a prospective study. Gastrointest Endosc. (2004) 60(5):721–31. 10.1016/s0016-5107(04)02169-8 15557948

[69] Freeman ML Guda NM. Prevention of post-ERCP pancreatitis: a comprehensive review. Gastrointest Endosc. (2004) 59(7):845–64. 10.1016/s0016-5107(04)00353-0 15173799

[70] Freeman ML Overby C Qi DF. Pancreatic stent insertion: consequences of failure and results of a modified technique to maximize success. Gastrointest Endosc. (2004) 59(1):8–14. 10.1016/s0016-5107(03)02530-6 14722540

[71] Mallery S Matlock J Freeman ML. EUS-Guided rendezvous drainage of obstructed biliary and pancreatic ducts: report of 6 cases. Gastrointest Endosc. (2004) 59(1):100–7. 10.1016/s0016-5107(03)02300-9 14722561

[72] Park MS Kim TK Kim KW Park SW Lee JK Kim JS Differentiation of extrahepatic bile duct cholangiocarcinoma from benign stricture: findings at MRCP versus ERCP. Radiology. (2004) 233(1):234–40. 10.1148/radiol.2331031446 15333766

[73] Rosch T Hofrichter K Frimberger E Meining A Born P Weigert N ERCP or EUS for tissue diagnosis of biliary strictures? A prospective comparative study. Gastrointest Endosc. (2004) 60(3):390–6. 10.1016/s0016-5107(04)01732-8 15332029

[74] Sahani DV Kalva SP Farrell J Maher MM Saini S Mueller PR Autoimmune pancreatitis: imaging features. Radiology. (2004) 233(2):345–52. 10.1148/radiol.2332031436 15459324

[75] Singh P Das A Isenberg G Wong RCK Sivak MV Agrawal D Does prophylactic pancreatic stent placement reduce the risk of post-ERCP acute pancreatitis? A meta-analysis of controlled trials. Gastrointest Endosc. (2004) 60(4):544–50. 10.1016/s0016-5107(04)02013-9 15472676

[76] Adler DG Baron TH Davila RE Egan J Hirota WK Leighton JA ASGE guideline: the role of ERCP in diseases of the biliary tract and the pancreas. Gastrointest Endosc. (2005) 62(1):1–8. 10.1016/j.gie.2005.04.015 15990812

[77] Freeman ML. ERCP cannulation: a review of reported techniques. Gastrointest Endosc. (2005) 61(1):112–25. 10.1016/s0016-5107(04)02463-0 15672074

[78] Riphaus A Stergiou N Wehrmann T. Sedation with propofol for routine ERCP in high-risk octogenarians: a randomized, controlled study. Am J Gastroenterol. (2005) 100(9):1957–63. 10.1111/j.1572-0241.2005.41672.x 16128939

[79] Cheng CL Sherman S Watkins JL Barnett J Freeman M Geenen J Risk factors for post-ERCP pancreatitis: a prospective multicenter study. Am J Gastroenterol. (2006) 101(1):139–47. 10.1111/j.1572-0241.2006.00380.x 16405547

[80] Kahaleh M Hernandez AJ Tokar J Adams RB Shami VM Yeaton P. Interventional EUS-guided cholangiography: evaluation of a technique in evolution. Gastrointest Endosc. (2006) 64(1):52–9. 10.1016/j.gie.2006.01.063 16813803

[81] Martin DJ Vernon DR Toouli J. Surgical versus endoscopic treatment of bile duct stones. Cochrane Database Syst Rev. (2006) 19(2):62. 10.1002/14651858.CD003327.pub2 16625577

[82] Verdonk RC Buis CI Porte RJ Van der Jagt EJ Limburg AJ Van den Berg AP Anastomotic biliary strictures after liver transplantation: causes and consequences. Liver Transplant. (2006) 12(5):726–35. 10.1002/lt.20714 16628689

[83] Bories E Pesenti C Caillol F Lopes C Giovannini M. Transgastric endoscopic ultrasonography-guided biliary drainage: results of a pilot study. Endoscopy. (2007) 39(4):287–91. 10.1055/s-2007-966212 17357952

[84] Williams EJ Taylor S Fairclough P Hamlyn A Logan RF Martin D Are we meeting the standards set for endoscopy? Results of a large-scale prospective survey of endoscopic retrograde cholangio-pancreatograph practice. Gut. (2007) 56(6):821–9. 10.1136/gut.2006.097543 17145737 PMC1954883

[85] Williams EJ Taylor S Fairclough P Hamlyn A Logan RF Martin D Risk factors for complication following ERCP; results of a large-scale, prospective multicenter study. Endoscopy. (2007) 39(9):793–801. 10.1055/s-2007-966723 17703388

[86] Bailey AA Bourke MJ Williams SJ Walsh PR Murray MA Lee EYT A prospective randomized trial of cannulation technique in ERCP: effects on technical success and post-ERCP pancreatitis. Endoscopy. (2008) 40(4):296–301. 10.1055/s-2007-995566 18389448

[87] Elmunzer BJ Waljee AK Elta GH Taylor JR Fehmi SMA Higgins PDR. A meta-analysis of rectal nsaids in the prevention of post-ERCP pancreatitis. Gut. (2008) 57(9):1262–7. 10.1136/gut.2007.140756 18375470

[88] Kahaleh M Behm B Clarke BW Brock A Shami VM De La Rue SA Temporary placement of covered self-expandable metal stents in benign biliary strictures: a new paradigm? (with video). Gastrointest Endosc. (2008) 67(3):446–54. 10.1016/j.gie.2007.06.057 18294506

[89] Williams EJ Green J Beckingham I Parks R Martin D Lombard M. Guidelines on the management of common bile duct stones (CBDS). Gut. (2008) 57(7):1004–21. 10.1136/gut.2007.121657 18321943

[90] Cotton PB Garrow DA Gallagher J Romagnuolo J. Risk factors for complications after ERCP: a multivariate analysis of 11,497 procedures over 12 years. Gastrointest Endosc. (2009) 70(1):80–8. 10.1016/j.gie.2008.10.039 19286178

[91] Wang P Li ZS Liu F Ren X Lu NH Fan ZN Risk factors for ERCP-related complications: a prospective multicenter study. Am J Gastroenterol. (2009) 104(1):31–40. 10.1038/ajg.2008.5 19098846

[92] Dumonceau JM Andriulli A Deviere J Mariani A Rigaux J Baron TH European society of gastrointestinal endoscopy (ESGE) guideline: prophylaxis of post-ERCP pancreatitis. Endoscopy. (2010) 42(6):503–15. 10.1055/s-0029-1244208 20506068

[93] Kullman E Frozanpor F Soderlund C Linder S Sandstrom P Lindhoff-Larsson A Covered versus uncovered self-expandable nitinol stents in the palliative treatment of malignant distal biliary obstruction: results from a randomized, multicenter study. Gastrointest Endosc. (2010) 72(5):915–23. 10.1016/j.gie.2010.07.036 21034892

[94] Rogers SJ Cello JP Horn JK Siperstein AE Schecter WP Campbell AR Prospective randomized trial of lc plus lcbde vs ERCP/S plus lc for common bile duct stone disease. Arch Surg. (2010) 145(1):28–33. 10.1001/archsurg.2009.226 20083751

[95] Testoni PA Mariani A Giussani A Vailati C Masci E Macarri G Risk factors for post-ERCP pancreatitis in high- and low-volume centers and among expert and non-expert operators: a prospective multicenter study. Am J Gastroenterol. (2010) 105(8):1753–61. 10.1038/ajg.2010.136 20372116

[96] van der Gaag NA Rauws EAJ van Eijck CHJ Bruno MJ van der Harst E Kubben F Preoperative biliary drainage for cancer of the head of the pancreas. N Engl J Med. (2010) 362(2):129–37. 10.1056/NEJMoa0903230 20071702

[97] Choudhary A Bechtold ML Arif M Szary NM Puli SR Othman MO Pancreatic stents for prophylaxis against post-ERCP pancreatitis: a meta-analysis and systematic review. Gastrointest Endosc. (2011) 73(2):275–82. 10.1016/j.gie.2010.10.039 21295641

[98] Park DH Jang JW Lee SS Seo DW Lee SK Kim MH. EUS-guided biliary drainage with transluminal stenting after failed ERCP: predictors of adverse events and long-term results. Gastrointest Endosc. (2011) 74(6):1276–84. 10.1016/j.gie.2011.07.054 21963067

[99] Anderson MA Fisher L Jain R Evans JA Appalaneni V Ben-Menachem T Complications of ERCP. Gastrointest Endosc. (2012) 75(3):467–73. 10.1016/j.gie.2011.07.010 22341094

[100] Dumonceau JM Delhaye M Tringali A Dominguez-Munoz JE Poley JW Arvanitaki M Endoscopic treatment of chronic pancreatitis: european society of gastrointestinal endoscopy (ESGE) clinical guideline. Endoscopy. (2012) 44(8):784–96. 10.1055/s-0032-1309840 22752888

[101] Elmunzer BJ Scheiman JM Lehman GA Chak A Mosler P Higgins PDR A randomized trial of rectal indomethacin to prevent post-ERCP pancreatitis. N Engl J Med. (2012) 366(15):1414–22. 10.1056/NEJMoa1111103 22494121 PMC3339271

[102] Shah RJ Smolkin M Yen R Ross A Kozarek RA Howell DA A multicenter, U.S. Experience of single-balloon, double-balloon, and rotational overtube-assisted enteroscopy ERCP in patients with surgically altered pancreaticobiliary anatomy. Gastrointest Endosc. (2013) 77(4):593–600. 10.1016/j.gie.2012.10.015 23290720

[103] Dumonceau JM Andriulli A Elmunzer BJ Mariani A Meister T Deviere J Prophylaxis of post-ERCP pancreatitis: european society of gastrointestinal endoscopy (ESGE) guideline—updated June 2014. Endoscopy. (2014) 46(9):798–814. 10.1055/s-0034-1377875 25148137

[104] Navaneethan U Njei B Lourdusamy V Konjeti R Vargo JJ Parsi MA. Comparative effectiveness of biliary brush cytology and intraductal biopsy for detection of malignant biliary strictures: a systematic review and meta-analysis. Gastrointest Endosc. (2015) 81(1):168–76. 10.1016/j.gie.2014.09.017 25440678 PMC4824293

[105] Testoni PA Mariani A Aabakken L Arvanitakis M Bories E Costamagna G Papillary cannulation and sphincterotomy techniques at Ercp: european society of gastrointestinal endoscopy (ESGE) clinical guideline. Endoscopy. (2016) 48(7):657–83. 10.1055/s-0042-108641 27299638

[106] Chandrasekhara V Khashab MA Muthusamy VR Acosta RD Agrawal D Bruining DH Adverse events associated with ERCP. Gastrointest Endosc. (2017) 85(1):32–47. 10.1016/j.gie.2016.06.051 27546389

[107] Sharaiha RZ Khan MA Kamal F Tyberg A Tombazzi CR Ali B Efficacy and safety of EUS-guided biliary drainage in comparison with percutaneous biliary drainage when ERCP fails: a systematic review and meta-analysis. Gastrointest Endosc. (2017) 85(5):904–14. 10.1016/j.gie.2016.12.023 28063840

[108] Dumonceau JM Tringali A Papanikolaou IS Blero D Mangiavillano B Schmidt A Endoscopic biliary stenting: indications, choice of stents, and results: european society of gastrointestinal endoscopy (ESGE) clinical guideline—updated October 2017. Endoscopy. (2018) 50(9):910–30. 10.1055/a-0659-9864 30086596

[109] Tan S Huang W Gao L Ren Y Peng Y Tang X. The 100 top cited articles in the field of digestive endoscopy: from 1950 to 2017. Rev Esp Enferm Dig. (2020) 112(9):701–7. 10.17235/reed.2020.6828/2019 32875805

[110] He B Zhang P Cai Q Shi S Xie H Zhang Y The top 100 most cited articles on bronchoscopy: a bibliometric analysis. BMC Pulm Med. (2020) 20(1):229. 10.1186/s12890-020-01266-9 32854666 PMC7450920

[111] Karsli B Tekin SB. The top 100 most-cited articles on ankle arthroscopy: bibliometric analysis. J Foot Ankle Surg. (2021) 60(3):477–81. 10.1053/j.jfas.2020.08.028 33518508

[112] Oecd Data. Gross Domestic Spending on R&D. Available from: https://data.oecd.org/rd/gross-domestic-spending-on-r

[113] Zhu X Kong Q Niu X Chen L Ge C. Mapping intellectual structure and research performance for the nanoparticles in pancreatic cancer field. Int J Nanomedicine. (2020) 15:5503–16. 10.2147/IJN.S253599 32801702 PMC7415461

[114] Ding H Wu C Liao N Zhan Q Sun W Huang Y Radiomics in oncology: a 10-year bibliometric analysis. Front Oncol. (2021) 11:689802. 10.3389/fonc.2021.689802 34616671 PMC8488302

[115] Garfield E. The history and meaning of the journal impact factor. Jama. (2006) 295(1):90–3. 10.1001/jama.295.1.90 16391221

[116] Paik WH Lee TH Park DH Choi JH Kim SO Jang S EUS-Guided biliary drainage versus ERCP for the primary palliation of malignant biliary obstruction: a multicenter randomized clinical trial. Am J Gastroenterol. (2018) 113(7):987–97. 10.1038/s41395-018-0122-8 29961772

[117] Akshintala VS Sperna Weiland CJ Bhullar FA Kamal A Kanthasamy K Kuo A Non-Steroidal anti-inflammatory drugs, intravenous fluids, pancreatic stents, or their combinations for the prevention of post-endoscopic retrograde cholangiopancreatography pancreatitis: a systematic review and network meta-analysis. Lancet Gastroenterol Hepatol. (2021) 6(9):733–42. 10.1016/S2468-1253(21)00170-9 34214449

[118] Classen M Deming L. Endoscopic sphincterotomy of the papilla of vater and extraction of stones from the choledochal ductt. Dtsch Med Wochenschr. (1974) 99:496–7. 10.1055/s-0028-1107790 4835515

[119] Yachimski PS Ross A. The future of endoscopic retrograde cholangiopancreatography. Gastroenterology. (2017) 153(2):338–44. 10.1053/j.gastro.2017.06.015 28647354

[120] Scheiman JM Carlos RC Barnett JL Elta GH Nostrant TT Chey WD Can endoscopic ultrasound or magnetic resonance cholangiopancreatography replace ERCP in patients with suspected biliary disease? A prospective trial and cost analysis. Am J Gastroenterol. (2001) 96(10):2900–4. 10.1111/j.1572-0241.2001.04245.x 11693324

[121] Kaltenthaler EC Walters SJ Chilcott J Blakeborough A Vergel YB Thomas S. MRCP compared to diagnostic ERCP for diagnosis when biliary obstruction is suspected: a systematic review. BMC Med Imaging. (2006) 6:9. 10.1186/1471-2342-6-9 16907974 PMC1579209

[122] Hekimoglu K Ustundag Y Dusak A Erdem Z Karademir B Aydemir S MRCP vs. ERCP in the evaluation of biliary pathologies: review of current literature. J Dig Dis. (2008) 9(3):162–9. 10.1111/j.1751-2980.2008.00339.x 18956595

[123] Griffin N Wastle ML Dunn WK Ryder SD Beckingham IJ. Magnetic resonance cholangiopancreatography versus endoscopic retrograde cholangiopancreatography in the diagnosis of choledocholithiasis. Eur J Gastroenterol Hepatol. (2003) 15(7):809–13. 10.1097/01.meg.0000059156.68845.46 12811312

[124] Park DH Kim MH Lee SK Lee SS Choi JS Lee YS Can MRCP replace the diagnostic role of ERCP for patients with choledochal cysts? Gastrointest Endosc. (2005) 62(3):360–6. 10.1016/j.gie.2005.04.026 16111952

[125] Aydelotte JD Ali J Huynh PT Coopwood TB Uecker JM Brown CV. Use of magnetic resonance cholangiopancreatography in clinical practice: not as good as we once thought. J Am Coll Surg. (2015) 221(1):215–9. 10.1016/j.jamcollsurg.2015.01.060 26047762

