# Supplementary material for: One hundred most cited articles related to Endoscopic retrograde cholangiopancreatography: A bibliometric analysis
Source: Front Surg. 2022 Nov 9;9:1005771. doi: 10.3389/fsurg.2022.1005771 (PMC9681810; doi:10.3389/fsurg.2022.1005771)
Supplement: Supplementary file 1 [file Table1.docx]

Supplementary table 1 The 100 most-cited papers in ERCP field.

| Rank | Article title | Times cited, WOS core | Publication year | Average per year |
| --- | --- | --- | --- | --- |
| 1 | Complications of endoscopic biliary sphincterotomy | 1925 | 1996 | 71.3 |
| 2 | Major early complications from diagnostic and therapeutic ERCP: a prospective multicenter study | 898 | 1998 | 35.92 |
| 3 | Risk factors for post-ERCP pancreatitis: a prospective, multicenter study | 844 | 2001 | 38.36 |
| 4 | CONTROLLED TRIAL OF URGENT ENDOSCOPIC RETROGRADE CHOLANGIOPANCREATOGRAPHY AND ENDOSCOPIC SPHINCTEROTOMY VERSUS CONSERVATIVE TREATMENT FOR ACUTE-PANCREATITIS DUE TO GALLSTONES | 662 | 1988 | 18.91 |
| 5 | Complications of diagnostic and therapeutic ERCP: A prospective multicenter study | 656 | 2001 | 29.82 |
| 6 | Incidence rates of post-ERCP complications: A systematic survey of prospective studies | 652 | 2007 | 40.75 |
| 7 | Preoperative Biliary Drainage for Cancer of the Head of the Pancreas | 602 | 2010 | 46.31 |
| 8 | EARLY TREATMENT OF ACUTE BILIARY PANCREATITIS BY ENDOSCOPIC PAPILLOTOMY | 567 | 1993 | 18.9 |
| 9 | Risk factors for complications after ERCP: a multivariate analysis of 11,497 procedures over 12 years | 433 | 2009 | 30.93 |
| 10 | Risk factors for complications after performance of ERCP | 419 | 2002 | 19.95 |
| 11 | Risk factors for post-ERCP pancreatitis: A prospective multicenter study | 418 | 2006 | 24.59 |
| 12 | Early ERCP and papillotomy compared with conservative treatment for acute biliary pancreatitis | 418 | 1997 | 16.08 |
| 13 | A Randomized Trial of Rectal Indomethacin to Prevent Post-ERCP Pancreatitis | 414 | 2012 | 37.64 |
| 14 | Endoscopic ultrasound-guided bilioduodenal anastomosis: A new technique for biliary drainage | 405 | 2001 | 18.41 |
| 15 | Complications of cholecystectomy: Risks of the laparoscopic approach and protective effects of operative cholangiography - A population-based study | 360 | 1999 | 15 |
| 16 | Guidelines on the management of common bile duct stones (CBDS) | 354 | 2008 | 23.6 |
| 17 | Prophylaxis of post-ERCP pancreatitis: European Society of Gastrointestinal Endoscopy (ESGE) Guideline - Updated June 2014 | 337 | 2014 | 37.44 |
| 18 | Randomised trial of laparoscopic exploration of common bile duct versus postoperative endoscopic retrograde cholangiography for common bile duct stones | 336 | 1998 | 13.44 |
| 19 | Prevention of post-ERCP pancreatitis: a comprehensive review | 329 | 2004 | 17.32 |
| 20 | Complications of ERCP: a prospective study | 311 | 2004 | 16.37 |
| 21 | Gabexate for the prevention of pancreatic damage related to endoscopic retrograde cholangiopancreatography | 309 | 1996 | 11.44 |
| 22 | Endosonography-guided fine needle aspiration biopsy in the evaluation of pancreatic masses | 304 | 2002 | 14.48 |
| 23 | Adverse events associated with ERCP | 297 | 2017 | 49.5 |
| 24 | Pancreatic stenting prevents pancreatitis after biliary sphincterotomy in patients with sphincter of oddi dysfunction | 296 | 1998 | 11.84 |
| 25 | Randomised trial of endoscopic balloon dilation versus endoscopic sphincterotomy for removal of bileduct stones | 293 | 1997 | 11.27 |
| 26 | Risk Factors for ERCP-Related Complications: A Prospective Multicenter Study | 292 | 2009 | 20.86 |
| 27 | Risk factors for pancreatitis following endoscopic retrograde cholangiopancreatography: A meta-analysis | 280 | 2003 | 14 |
| 28 | ASGE guideline: the role of ERCP in diseases of the biliary tract and the pancreas | 272 | 2005 | 15.11 |
| 29 | Autoimmune pancreatitis: Imaging features | 271 | 2004 | 14.26 |
| 30 | Complications of ERCP | 266 | 2012 | 24.18 |
| 31 | VALUE OF ENDOBILIARY BRUSH CYTOLOGY AND BIOPSIES FOR THE DIAGNOSIS OF MALIGNANT BILE-DUCT STENOSIS - RESULTS OF A PROSPECTIVE-STUDY | 265 | 1995 | 9.46 |
| 32 | EUS-guided rendezvous drainage of obstructed biliary and pancreatic ducts: report of 6 cases | 263 | 2004 | 13.84 |
| 33 | ERCP-SCOPIC AND ENDOSCOPIC SPHINCTEROTOMY-INDUCED PANCREATITIS | 254 | 1991 | 7.94 |
| 34 | PROSPECTIVE EVALUATION OF ENDOSCOPIC ULTRASONOGRAPHY AND ENDOSCOPIC RETROGRADE CHOLANGIOPANCREATOGRAPHY IN PATIENTS WITH CHRONIC ABDOMINAL-PAIN OF SUSPECTED PANCREATIC ORIGIN | 248 | 1993 | 8.27 |
| 35 | Risk factors for complication following ERCP; results of a large-scale, prospective multicenter study | 245 | 2007 | 15.31 |
| 36 | Magnetic resonance cholangiography: Comparison with endoscopic retrograde cholangiopancreatography | 244 | 1996 | 9.04 |
| 37 | Does prophylactic pancreatic stent placement reduce the risk of post-ERCP acute pancreatitis? A meta-analysis of controlled trials | 242 | 2004 | 12.74 |
| 38 | Prospective assessment of the ability of endoscopic ultrasound to diagnose, exclude, or establish the severity of chronic pancreatitis found by endoscopic retrograde cholangiopancreatography | 239 | 1998 | 9.56 |
| 39 | Quantitative assessment of procedural competence - A prospective study of training in endoscopic retrograde cholangiopancreatography | 238 | 1996 | 8.81 |
| 40 | Papillary cannulation and sphincterotomy techniques at ERCP: European Society of Gastrointestinal Endoscopy (ESGE) Clinical Guideline | 236 | 2016 | 33.71 |
| 41 | Management of duodenal perforation after endoscopic retrograde cholangiopancreatography and sphincterotomy | 235 | 2000 | 10.22 |
| 42 | ENDOSCOPIC SNARE EXCISION OF BENIGN ADENOMAS OF THE PAPILLA OF VATER | 234 | 1993 | 7.8 |
| 43 | Prospective evaluation of endoscopic ultrasonography, endoscopic retrograde pancreatography, and secretin test in the diagnosis of chronic pancreatitis | 232 | 1998 | 9.28 |
| 44 | Does a pancreatic duct stent prevent post-ERCP pancreatitis? A prospective randomized study | 231 | 2003 | 11.55 |
| 45 | Endoscopic management of adenoma of the major duodenal papilla | 229 | 2004 | 12.05 |
| 46 | Triple-tissue sampling at ERCP in malignant biliary obstruction | 224 | 2000 | 9.74 |
| 47 | Endoscopic balloon dilation of the biliary sphincter compared to endoscopic biliary sphincterotomy for removal of common bile duct stones during ERCP: A metaanalysis of randomized, controlled trials | 220 | 2004 | 11.58 |
| 48 | ERCP or EUS for tissue diagnosis of biliary strictures? A prospective comparative study | 220 | 2004 | 11.58 |
| 49 | ERCP cannulation: a review of reported techniques | 219 | 2005 | 12.17 |
| 50 | Biliary tract complications after orthotopic liver transplantation with choledochocholedochostomy anastomosis: endoscopic findings and results of therapy | 218 | 2002 | 10.38 |
| 51 | Pancreas divisum: Evaluation with MR cholangiopancreatography | 218 | 1996 | 8.07 |
| 52 | Comparative effectiveness of biliary brush cytology and intraductal biopsy for detection of malignant biliary strictures: a systematic review and meta-analysis | 217 | 2015 | 27.13 |
| 53 | Anastomotic biliary strictures after liver transplantation: Causes and consequences | 212 | 2006 | 12.47 |
| 54 | Endoscopic pancreatic stent drainage in chronic pancreatitis and a dominant stricture: Long-term results | 210 | 1995 | 7.5 |
| 55 | Endoscopic biliary stenting: indications, choice of stents, and results: European Society of Gastrointestinal Endoscopy (ESGE) Clinical Guideline - Updated October 2017 | 208 | 2018 | 41.6 |
| 56 | European Society of Gastrointestinal Endoscopy (ESGE) Guideline: Prophylaxis of post-ERCP pancreatitis | 207 | 2010 | 15.92 |
| 57 | Interventional EUS-guided cholangiography: evaluation of a technique in evolution | 207 | 2006 | 12.18 |
| 58 | Efficacy and safety of intravenous propofol sedation during routine ERCP: a prospective, controlled study | 206 | 1999 | 8.58 |
| 59 | Incidence, severity, and mortality of post-ERCP pancreatitis: a systematic review by using randomized, controlled trials | 201 | 2015 | 25.13 |
| 60 | Covered versus uncovered self-expandable nitinol stents in the palliative treatment of malignant distal biliary obstruction: results from a randomized, multicenter study | 197 | 2010 | 15.15 |
| 61 | ENDOSCOPIC RETROGRADE FORCEPS BIOPSY AND BRUSH CYTOLOGY OF BILIARY STRICTURES - A PROSPECTIVE-STUDY | 197 | 1995 | 7.04 |
| 62 | ERCP in patients with long-limb Roux-en-Y gastrojejunostomy and intact papilla | 196 | 2002 | 9.33 |
| 63 | Diclofenac reduces the incidence of acute pancreatitis after endoscopic retrograde cholanglopancreatography | 194 | 2003 | 9.7 |
| 64 | Choledocholithiasis: Comparison of MR cholangiography and endoscopic retrograde cholangiography | 193 | 1996 | 7.15 |
| 65 | Endoscopic treatment of chronic pancreatitis: European Society of Gastrointestinal Endoscopy (ESGE) Clinical Guideline | 192 | 2012 | 17.45 |
| 66 | Are we meeting the standards set for endoscopy? Results of a large-scale prospective survey of endoscopic retrograde cholangio-pancreatograph practice | 192 | 2007 | 12 |
| 67 | ERCP-related perforations: Risk factors and management | 190 | 2002 | 9.05 |
| 68 | Prospective Randomized Trial of LC plus LCBDE vs ERCP/S plus LC for Common Bile Duct Stone Disease | 188 | 2010 | 14.46 |
| 69 | Endosonography-guided cholangiopancreatography | 187 | 1996 | 6.93 |
| 70 | Risk Factors for Post-ERCP Pancreatitis in High- and Low-Volume Centers and Among Expert and Non-Expert Operators: A Prospective Multicenter Study | 184 | 2010 | 14.15 |
| 71 | A prospective comparison of the diagnostic accuracy of ERCP, MRCP, CT, and EUS in biliary strictures | 184 | 2002 | 8.76 |
| 72 | Endoscopic management of postoperative biliary complications in orthotopic liver transplantation | 184 | 2000 | 8 |
| 73 | Natural history of primary sclerosing cholangitis and prognostic value of cholangiography in a Dutch population | 183 | 2002 | 8.71 |
| 74 | Long-term outcome in patients with benign biliary strictures treated endoscopically with multiple stents | 182 | 2002 | 8.67 |
| 75 | Adverse outcomes of ERCP | 182 | 2002 | 8.67 |
| 76 | EUS-guided biliary drainage with transluminal stenting after failed ERCP: predictors of adverse events and long-term results | 181 | 2011 | 15.08 |
| 77 | Intraductal papillary and mucinous tumors of the pancreas: accuracy of preoperative computed tomography, endoscopic retrograde pancreatography and endoscopic ultrasonography, and long-term outcome in a large surgical series | 181 | 1998 | 7.24 |
| 78 | PROGRESSIVE LOSS OF PANCREATIC FUNCTION IN CHRONIC-PANCREATITIS IS DELAYED BY MAIN PANCREATIC DUCT DECOMPRESSION - A LONGITUDINAL PROSPECTIVE ANALYSIS OF THE MODIFIED PUESTOW PROCEDURE | 179 | 1993 | 5.97 |
| 79 | ENDOSCOPIC BALLOON SPHINCTEROPLASTY (PAPILLARY DILATION) FOR BILE-DUCT STONES - EFFICACY, SAFETY, AND FOLLOW-UP IN 100 PATIENTS | 178 | 1995 | 6.36 |
| 80 | Association of preoperative biliary stenting with increased postoperative infectious complications in proximal cholangiocarcinoma | 177 | 1999 | 7.38 |
| 81 | Efficacy and safety of EUS-guided biliary drainage in comparison with percutaneous biliary drainage when ERCP fails: a systematic review and meta-analysis | 176 | 2017 | 29.33 |
| 82 | A multicenter, U.S. experience of single-balloon, double-balloon, and rotational overtube-assisted enteroscopy ERCP in patients with surgically altered pancreaticobiliary anatomy | 176 | 2013 | 17.6 |
| 83 | Pancreatic stents for prophylaxis against post-ERCP pancreatitis: a meta-analysis and systematic review | 175 | 2011 | 14.58 |
| 84 | Surgical versus endoscopic treatment of bile duct stones | 175 | 2006 | 10.29 |
| 85 | A meta-analysis of rectal NSAIDs in the prevention of post-ERCP pancreatitis | 174 | 2008 | 11.6 |
| 86 | National Institutes of Health State-of-the-Science Conference Statement: ERCP for diagnosis and therapy, January 14-16, 2002 | 173 | 2002 | 8.24 |
| 87 | Temporary placement of covered self-expandable metal stents in benign biliary strictures: a new paradigm? (with video) | 172 | 2008 | 11.47 |
| 88 | ERCP features in 27 patients with autoimmune pancreatitis | 171 | 2002 | 8.14 |
| 89 | A prospective evaluation of cytology from biliary strictures | 170 | 1997 | 6.54 |
| 90 | Long-term follow-up after endoscopic sphincterotomy for bile duct stones in patients younger than 60 years of age | 170 | 1996 | 6.3 |
| 91 | A prospective randomized trial of cannulation technique in ERCP: effects on technical success and post-ERCP pancreatitis | 168 | 2008 | 11.2 |
| 92 | Transgastric endoscopic ultrasonography-guided biliary drainage: results of a pilot study | 168 | 2007 | 10.5 |
| 93 | Endoscopic ultrasonography versus cholangiography for the diagnosis of choledocholithiasis | 168 | 1998 | 6.72 |
| 94 | Pancreatic cancer detection with magnetic resonance cholangiopancreatography and endoscopic retrograde cholangiopancreatography: a prospective controlled study | 165 | 2000 | 7.17 |
| 95 | Endoscopic management of common bile duct stones: European Society of Gastrointestinal Endoscopy (ESGE) guideline | 164 | 2019 | 41 |
| 96 | Papillary dilation vs sphincterotomy in endoscopic removal of bile duct stones - A randomized trial with manometric function | 163 | 1995 | 5.82 |
| 97 | ERCP-related adverse events: European Society of Gastrointestinal Endoscopy (ESGE) Guideline | 160 | 2020 | 53.33 |
| 98 | Differentiation of extrahepatic bile duct cholangiocarcinoma from benign stricture: Findings at MRCP versus ERCP | 160 | 2004 | 8.42 |
| 99 | Sedation with propofol for routine ERCP in high-risk octogenarians: A randomized, controlled study | 159 | 2005 | 8.83 |
| 100 | Pancreatic stent insertion: consequences of failure and results of a modified technique to maximize success | 159 | 2004 | 8.37 |
