# Supplementary material for: One hundred most cited articles related to Endoscopic retrograde cholangiopancreatography: A bibliometric analysis
Source: Front Surg. 2022 Nov 9;9:1005771. doi: 10.3389/fsurg.2022.1005771 (PMC9681810; doi:10.3389/fsurg.2022.1005771)
Supplement: Supplementary file 2 [file Table2.docx]

| label | replace by |
| --- | --- |
| bile duct stone | bile-duct stones |
| biliary obstruction | bile-duct obstruction |
| billroth ii gastrectomy | billroth-ii gastrectomy |
| carcinoma | cancer |
| common duct | common bile-duct |
| disease | diseases |
| endoscopic retrograde cholangiopancreatography (ercp) | ercp |
| endoscopic retrograde cholangiopancreatography | ercp |
| endoscopic ultrasound | endoscopic ultrasonography |
| expanding metal stents | expandable metal stents |
| endoscopic sphincterotomy techniques | endoscopic sphincterotomy |
| gallstone disease | gallstones |
| long-term efficacy | long-term outcomes |
| long-term | long-term outcomes |
| magnetic resonance (mr), cholangiopancreatography | magnetic-resonance cholangiopancreatography |
| malignancy | cancer |
| mr-cholangiopancreatography | magnetic-resonance cholangiopancreatography |
| mr cholangiography | magnetic-resonance cholangiography |
| necrotizing pancreatitis | acute necrotizing pancreatitis |
| pancreatic carcinoma | pancreatic-cancer |
| pancreatic-stent placement | pancreatic stent placement |
| papillary-mucinous tumor | papillary mucinous neoplasms |
| randomized controlled-trial | randomized controlled-trials |
| randomized-trial | randomized controlled-trials |
| recurrence | recurrent |
| retrograde cholangiopancreatography | ercp |
| retrograde cholangiopancreatography ercp | ercp |
| retrograde cholangiopancreatography pancreatitis | post-ercp pancreatitis |
| risk-factor | risk-factors |
| risk | risk-factors |
| surgical treatment | surgical-treatment |
| surgical-management | surgical-treatment |
| term follow-up | term-follow-up |
| tumors | cancer |
| villous adenoma | villous tumors |
| ultrasound | ultrasonography |
| ct | computed-tomography |
