# Supplementary material for: One hundred most cited articles related to Endoscopic retrograde cholangiopancreatography: A bibliometric analysis
Source: Front Surg. 2022 Nov 9;9:1005771. doi: 10.3389/fsurg.2022.1005771 (PMC9681810; doi:10.3389/fsurg.2022.1005771)
Supplement: Supplementary file 4 [file Table4.docx]

Supplementary table 1 The 100 most-cited papers in the ERCP field.

* “N/A” was assigned when the journal impact factor was not available or had not been assigned in the year of publication

| Rank | Article title | Times cited, WOS core | Publication year | Citations per year | Open Access or not | Impact factor in the year of publication * |
| --- | --- | --- | --- | --- | --- | --- |
| 1 | Complications of endoscopic biliary sphincterotomy(28) | 1925 | 1996 | 71.3 | NO | N/A |
| 2 | Major early complications from diagnostic and therapeutic ERCP: a prospective multicenter study(38) | 898 | 1998 | 35.92 | NO | 3.531 |
| 3 | Risk factors for post-ERCP pancreatitis: a prospective, multicenter study | 844 | 2001 | 38.36 | YES | 2.776 |
| 4 | CONTROLLED TRIAL OF URGENT ENDOSCOPIC RETROGRADE(49) CHOLANGIOPANCREATOGRAPHY AND ENDOSCOPIC SPHINCTEROTOMY VERSUS CONSERVATIVE TREATMENT FOR ACUTE-PANCREATITIS DUE TO GALLSTONES(13) | 662 | 1988 | 18.91 | NO | 11.793 |
| 5 | Complications of diagnostic and therapeutic ERCP: A prospective multicenter study(51) | 656 | 2001 | 29.82 | NO | 3.549 |
| 6 | Incidence rates of post-ERCP complications: A systematic survey of prospective studies(83) | 652 | 2007 | 40.75 | NO | 6.101 |
| 7 | Preoperative Biliary Drainage for Cancer of the Head of the Pancreas(97) | 602 | 2010 | 46.31 | NO | 53.486 |
| 8 | EARLY TREATMENT OF ACUTE BILIARY PANCREATITIS BY ENDOSCOPIC PAPILLOTOMY(16) | 567 | 1993 | 18.9 | NO | N/A |
| 9 | Risk factors for complications after ERCP: a multivariate analysis of 11,497 procedures over 12 years(91) | 433 | 2009 | 30.93 | NO | 6.713 |
| 10 | Risk factors for complications after performance of ERCP(61) | 419 | 2002 | 19.95 | NO | 3.037 |
| 11 | Risk factors for post-ERCP pancreatitis: A prospective multicenter study(79) | 418 | 2006 | 24.59 | YES | 5.608 |
| 12 | Early ERCP and papillotomy compared with conservative treatment for acute biliary pancreatitis(33) | 418 | 1997 | 16.08 | NO | 27.766 |
| 13 | A Randomized Trial of Rectal Indomethacin to Prevent Post-ERCP Pancreatitis(102) | 414 | 2012 | 37.64 | YES | 51.658 |
| 14 | Endoscopic ultrasound-guided bilioduodenal anastomosis: A new technique for biliary drainage(50) | 405 | 2001 | 18.41 | NO | 1.459 |
| 15 | Complications of cholecystectomy: Risks of the laparoscopic approach and protective effects of operative cholangiography - A population-based study(42) | 360 | 1999 | 15 | YES | 5.647 |
| 16 | Guidelines on the management of common bile duct stones (CBDS)(90) | 354 | 2008 | 23.6 | NO | 9.766 |
| 17 | Prophylaxis of post-ERCP pancreatitis: European Society of Gastrointestinal Endoscopy (ESGE) Guideline - Updated June 2014(104) | 337 | 2014 | 37.44 | YES | 5.104 |
| 18 | Randomised trial of laparoscopic exploration of common bile duct versus postoperative endoscopic retrograde cholangiography for common bile duct stones(39) | 336 | 1998 | 13.44 | NO | 11.793 |
| 19 | Prevention of post-ERCP pancreatitis: a comprehensive review(69) | 329 | 2004 | 17.32 | NO | 3.483 |
| 20 | Complications of ERCP: a prospective study(68) | 311 | 2004 | 16.37 | NO | 3.483 |
| 21 | Gabexate for the prevention of pancreatic damage related to endoscopic retrograde cholangiopancreatography(26) | 309 | 1996 | 11.44 | NO | N/A |
| 22 | Endosonography-guided fine needle aspiration biopsy in the evaluation of pancreatic masses(56) | 304 | 2002 | 14.48 | NO | 3.953 |
| 23 | Adverse events associated with ERCP(108) | 297 | 2017 | 49.5 | YES | 7.204 |
| 24 | Pancreatic stenting prevents pancreatitis after biliary sphincterotomy in patients with sphincter of oddi dysfunction(41) | 296 | 1998 | 11.84 | NO | 10.33 |
| 25 | Randomised trial of endoscopic balloon dilation versus endoscopic sphincterotomy for removal of bileduct stones(32) | 293 | 1997 | 11.27 | NO | 16.135 |
| 26 | Risk Factors for ERCP-Related Complications: A Prospective Multicenter Study(92) | 292 | 2009 | 20.86 | NO | 6.012 |
| 27 | Risk factors for pancreatitis following endoscopic retrograde cholangiopancreatography: A meta-analysis(64) | 280 | 2003 | 14 | NO | 3.227 |
| 28 | ASGE guideline: the role of ERCP in diseases of the biliary tract and the pancreas(76) | 272 | 2005 | 15.11 | NO | 3.923 |
| 29 | Autoimmune pancreatitis: Imaging features(74) | 271 | 2004 | 14.26 | NO | 5.076 |
| 30 | Complications of ERCP(100) | 266 | 2012 | 24.18 | NO | 5.21 |
| 31 | VALUE OF ENDOBILIARY BRUSH CYTOLOGY AND BIOPSIES FOR THE DIAGNOSIS OF MALIGNANT BILE-DUCT STENOSIS - RESULTS OF A PROSPECTIVE-STUDY(22) | 265 | 1995 | 9.46 | NO | N/A |
| 32 | EUS-guided rendezvous drainage of obstructed biliary and pancreatic ducts: report of 6 cases(71) | 263 | 2004 | 13.84 | NO | 3.483 |
| 33 | ERCP-SCOPIC AND ENDOSCOPIC SPHINCTEROTOMY-INDUCED PANCREATITIS | 254 | 1991 | 7.94 | NO | N/A |
| 34 | PROSPECTIVE EVALUATION OF ENDOSCOPIC ULTRASONOGRAPHY AND ENDOSCOPIC(14) RETROGRADE CHOLANGIOPANCREATOGRAPHY IN PATIENTS WITH CHRONIC ABDOMINAL-PAIN OF SUSPECTED PANCREATIC ORIGIN(18) | 248 | 1993 | 8.27 | NO | N/A |
| 35 | Risk factors for complication following ERCP; results of a large-scale, prospective multicenter study(86) | 245 | 2007 | 15.31 | NO | 4.166 |
| 36 | Magnetic resonance cholangiography: Comparison with endoscopic retrograde cholangiopancreatography(30) | 244 | 1996 | 9.04 | NO | N/A |
| 37 | Does prophylactic pancreatic stent placement reduce the risk of post-ERCP acute pancreatitis? A meta-analysis of controlled trials(75) | 242 | 2004 | 12.74 | NO | 3.483 |
| 38 | Prospective assessment of the ability of endoscopic ultrasound to diagnose, exclude, or establish the severity of chronic pancreatitis found by endoscopic retrograde cholangiopancreatography(40) | 239 | 1998 | 9.56 | NO | 3.531 |
| 39 | Quantitative assessment of procedural competence - A prospective study of training in endoscopic retrograde cholangiopancreatography(29) | 238 | 1996 | 8.81 | NO | N/A |
| 40 | Papillary cannulation and sphincterotomy techniques at ERCP: European Society of Gastrointestinal Endoscopy (ESGE) Clinical Guideline(107) | 236 | 2016 | 33.71 | YES | 6.107 |
| 41 | Management of duodenal perforation after endoscopic retrograde cholangiopancreatography and sphincterotomy(48) | 235 | 2000 | 10.22 | YES | 5.987 |
| 42 | ENDOSCOPIC SNARE EXCISION OF BENIGN ADENOMAS OF THE PAPILLA OF VATER(15) | 234 | 1993 | 7.8 | NO | N/A |
| 43 | Prospective evaluation of endoscopic ultrasonography, endoscopic retrograde pancreatography, and secretin test in the diagnosis of chronic pancreatitis(36) | 232 | 1998 | 9.28 | NO | 3.531 |
| 44 | Does a pancreatic duct stent prevent post-ERCP pancreatitis? A prospective randomized study(63) | 231 | 2003 | 11.55 | NO | 3.328 |
| 45 | Endoscopic management of adenoma of the major duodenal papilla(67) | 229 | 2004 | 12.05 | NO | 3.483 |
| 46 | Triple-tissue sampling at ERCP in malignant biliary obstruction(46) | 224 | 2000 | 9.74 | NO | 2.82 |
| 47 | Endoscopic balloon dilation of the biliary sphincter compared to endoscopic biliary sphincterotomy for removal of common bile duct stones during ERCP: A metaanalysis of randomized, controlled trials(66) | 220 | 2004 | 11.58 | NO | 4.716 |
| 48 | ERCP or EUS for tissue diagnosis of biliary strictures? A prospective comparative study(73) | 220 | 2004 | 11.58 | NO | 3.483 |
| 49 | ERCP cannulation: a review of reported techniques(77) | 219 | 2005 | 12.17 | NO | 3.923 |
| 50 | Biliary tract complications after orthotopic liver transplantation with choledochocholedochostomy anastomosis: endoscopic findings and results of therapy(59) | 218 | 2002 | 10.38 | NO | 3.037 |
| 51 | Pancreas divisum: Evaluation with MR cholangiopancreatography(25) | 218 | 1996 | 8.07 | NO | N/A |
| 52 | Comparative effectiveness of biliary brush cytology and intraductal biopsy for detection of malignant biliary strictures: a systematic review and meta-analysis(106) | 217 | 2015 | 27.13 | YES | 6.217 |
| 53 | Anastomotic biliary strictures after liver transplantation: Causes and consequences(82) | 212 | 2006 | 12.47 | YES | 4.629 |
| 54 | Endoscopic pancreatic stent drainage in chronic pancreatitis and a dominant stricture: Long-term results(19) | 210 | 1995 | 7.5 | NO | N/A |
| 55 | Endoscopic biliary stenting: indications, choice of stents, and results: European Society of Gastrointestinal Endoscopy (ESGE) Clinical Guideline - Updated October 2017(110) | 208 | 2018 | 41.6 | YES | 6.381 |
| 56 | European Society of Gastrointestinal Endoscopy (ESGE) Guideline: Prophylaxis of post-ERCP pancreatitis(93) | 207 | 2010 | 15.92 | YES | 6.096 |
| 57 | Interventional EUS-guided cholangiography: evaluation of a technique in evolution(80) | 207 | 2006 | 12.18 | NO | 4.825 |
| 58 | Efficacy and safety of intravenous propofol sedation during routine ERCP: a prospective, controlled study(44) | 206 | 1999 | 8.58 | NO | 3.225 |
| 59 | Incidence, severity, and mortality of post-ERCP pancreatitis: a systematic review by using randomized, controlled trials(105) | 201 | 2015 | 25.13 | NO | 6.217 |
| 60 | Covered versus uncovered self-expandable nitinol stents in the palliative treatment of malignant distal biliary obstruction: results from a randomized, multicenter study(94) | 197 | 2010 | 15.15 | NO | N/A |
| 61 | ENDOSCOPIC RETROGRADE FORCEPS BIOPSY AND BRUSH CYTOLOGY OF BILIARY STRICTURES - A PROSPECTIVE-STUDY(23) | 197 | 1995 | 7.04 | NO | 5.647 |
| 62 | ERCP in patients with long-limb Roux-en-Y gastrojejunostomy and intact papilla(62) | 196 | 2002 | 9.33 | NO | 3.037 |
| 63 | Diclofenac reduces the incidence of acute pancreatitis after endoscopic retrograde cholanglopancreatography(65) | 194 | 2003 | 9.7 | NO | 12.718 |
| 64 | Choledocholithiasis: Comparison of MR cholangiography and endoscopic retrograde cholangiography(27) | 193 | 1996 | 7.15 | NO | N/A |
| 65 | Endoscopic treatment of chronic pancreatitis: European Society of Gastrointestinal Endoscopy (ESGE) Clinical Guideline(101) | 192 | 2012 | 17.45 | YES | 5.735 |
| 66 | Are we meeting the standards set for endoscopy? Results of a large-scale prospective survey of endoscopic retrograde cholangio-pancreatograph practice(85) | 192 | 2007 | 12 | YES | 10.015 |
| 67 | ERCP-related perforations: Risk factors and management(54) | 190 | 2002 | 9.05 | NO | 1.7 |
| 68 | Prospective Randomized Trial of LC plus LCBDE vs ERCP/S plus LC for Common Bile Duct Stone Disease(95) | 188 | 2010 | 14.46 | YES | 4.5 |
| 69 | Endosonography-guided cholangiopancreatography(31) | 187 | 1996 | 6.93 | NO | N/A |
| 70 | Risk Factors for Post-ERCP Pancreatitis in High- and Low-Volume Centers and Among Expert and Non-Expert Operators: A Prospective Multicenter Study(96) | 184 | 2010 | 14.15 | NO | 6.882 |
| 71 | A prospective comparison of the diagnostic accuracy of ERCP, MRCP, CT, and EUS in biliary strictures(60) | 184 | 2002 | 8.76 | NO | 2.82 |
| 72 | Endoscopic management of postoperative biliary complications in orthotopic liver transplantation(47) | 184 | 2000 | 8 | NO | 3.037 |
| 73 | Natural history of primary sclerosing cholangitis and prognostic value of cholangiography in a Dutch population(58) | 183 | 2002 | 8.71 | YES | 6.323 |
| 74 | Long-term outcome in patients with benign biliary strictures treated endoscopically with multiple stents(53) | 182 | 2002 | 8.67 | NO | 3.037 |
| 75 | Adverse outcomes of ERCP(55) | 182 | 2002 | 8.67 | NO | 3.037 |
| 76 | EUS-guided biliary drainage with transluminal stenting after failed ERCP: predictors of adverse events and long-term results(99) | 181 | 2011 | 15.08 | NO | 3.531 |
| 77 | Intraductal papillary and mucinous tumors of the pancreas: accuracy of preoperative computed tomography, endoscopic retrograde pancreatography and endoscopic ultrasonography, and long-term outcome in a large surgical series(37) | 181 | 1998 | 7.24 | NO | 4.923 |
| 78 | PROGRESSIVE LOSS OF PANCREATIC FUNCTION IN CHRONIC-PANCREATITIS IS DELAYED BY MAIN PANCREATIC DUCT DECOMPRESSION - A LONGITUDINAL PROSPECTIVE ANALYSIS OF THE MODIFIED PUESTOW PROCEDURE(17) | 179 | 1993 | 5.97 | YES | N/A |
| 79 | ENDOSCOPIC BALLOON SPHINCTEROPLASTY (PAPILLARY DILATION) FOR BILE-DUCT STONES - EFFICACY, SAFETY, AND FOLLOW-UP IN 100 PATIENTS(20) | 178 | 1995 | 6.36 | NO | N/A |
| 80 | Association of preoperative biliary stenting with increased postoperative infectious complications in proximal cholangiocarcinoma(43) | 177 | 1999 | 7.38 | YES | 2.584 |
| 81 | Efficacy and safety of EUS-guided biliary drainage in comparison with percutaneous biliary drainage when ERCP fails: a systematic review and meta-analysis(109) | 176 | 2017 | 29.33 | NO | 4.9 |
| 82 | A multicenter, U.S. experience of single-balloon, double-balloon, and rotational overtube-assisted enteroscopy ERCP in patients with surgically altered pancreaticobiliary anatomy(103) | 176 | 2013 | 17.6 | NO | 7.204 |
| 83 | Pancreatic stents for prophylaxis against post-ERCP pancreatitis: a meta-analysis and systematic review(98) | 175 | 2011 | 14.58 | NO | N/A |
| 84 | Surgical versus endoscopic treatment of bile duct stones(81) | 175 | 2006 | 10.29 | NO | 4.923 |
| 85 | A meta-analysis of rectal NSAIDs in the prevention of post-ERCP pancreatitis(88) | 174 | 2008 | 11.6 | NO | 9.766 |
| 86 | National Institutes of Health State-of-the-Science Conference Statement: ERCP for diagnosis and therapy, January 14-16, 2002(52) | 173 | 2002 | 8.24 | NO | 3.037 |
| 87 | Temporary placement of covered self-expandable metal stents in benign biliary strictures: a new paradigm? (with video)(89) | 172 | 2008 | 11.47 | NO | 7.367 |
| 88 | ERCP features in 27 patients with autoimmune pancreatitis(57) | 171 | 2002 | 8.14 | NO | 3.037 |
| 89 | A prospective evaluation of cytology from biliary strictures(34) | 170 | 1997 | 6.54 | NO | N/A |
| 90 | Long-term follow-up after endoscopic sphincterotomy for bile duct stones in patients younger than 60 years of age(24) | 170 | 1996 | 6.3 | YES | 4.546 |
| 91 | A prospective randomized trial of cannulation technique in ERCP: effects on technical success and post-ERCP pancreatitis(87) | 168 | 2008 | 11.2 | NO | 4.166 |
| 92 | Transgastric endoscopic ultrasonography-guided biliary drainage: results of a pilot study(84) | 168 | 2007 | 10.5 | NO | 6.091 |
| 93 | Endoscopic ultrasonography versus cholangiography for the diagnosis of choledocholithiasis(35) | 168 | 1998 | 6.72 | NO | 3.531 |
| 94 | Pancreatic cancer detection with magnetic resonance cholangiopancreatography and endoscopic retrograde cholangiopancreatography: a prospective controlled study(45) | 165 | 2000 | 7.17 | NO | 10.232 |
| 95 | Endoscopic management of common bile duct stones: European Society of Gastrointestinal Endoscopy (ESGE) guideline(3) | 164 | 2019 | 41 | YES | 7.341 |
| 96 | Papillary dilation vs sphincterotomy in endoscopic removal of bile duct stones - A randomized trial with manometric function(21) | 163 | 1995 | 5.82 | NO | N/A |
| 97 | ERCP-related adverse events: European Society of Gastrointestinal Endoscopy (ESGE) Guideline(5) | 160 | 2020 | 53.33 | YES | 10.093 |
| 98 | Differentiation of extrahepatic bile duct cholangiocarcinoma from benign stricture: Findings at MRCP versus ERCP(72) | 160 | 2004 | 8.42 | NO | 5.076 |
| 99 | Sedation with propofol for routine ERCP in high-risk octogenarians: A randomized, controlled study(78) | 159 | 2005 | 8.83 | NO | 5.116 |
| 100 | Pancreatic stent insertion: consequences of failure and results of a modified technique to maximize success(70) | 159 | 2004 | 8.37 | NO | 3.483 |
